# Global spatiotemporal analysis of suicide epidemiology and risk factor associations from 2000 to 2019 using Bayesian space time hierarchical modeling

**DOI:** 10.1038/s41598-025-97064-6

**Published:** 2025-04-14

**Authors:** Chawarat Rotejanaprasert, Papin Thanutchapat, Chiraphat Phoncharoenwirot, Ornrakorn Mekchaiporn, Peerut Chienwichai, Richard J. Maude

**Affiliations:** 1 Department of Tropical Hygiene, Faculty of Tropical Medicine, Mahidol University, Bangkok, Thailand; 2 Mahidol-Oxford Tropical Medicine Research Unit, Faculty of Tropical Medicine, Mahidol University, Bangkok, Thailand; 3 Princess Srisavangavadhana College of Medicine, Chulabhorn Royal Academy, Bangkok, Thailand; 4 Department of Computer Engineering, Faculty of Engineering, King Mongkut’s University of Technology Thonburi, Bangkok, Thailand; 5 Centre for Tropical Medicine and Global Health, Nuffield Department of Medicine, University of Oxford, Oxford, UK; 6 The Open University, Milton Keynes, UK

**Keywords:** Suicide, Global, Spatiotemporal, Bayesian, Mental health, Health policy, Public health, Risk factors

## Abstract

Suicide is a significant global public health issue, with marked disparities in rates between countries. Much of the existing research has concentrated on high-income nations, creating a gap in the understanding of global suicide epidemiology. This study aims to address this gap through a comprehensive spatiotemporal analysis of global suicide trends from 2000 to 2019. Data were collected from the Global Health Observatory, encompassing 183 countries across five regions. Bayesian spatiotemporal modeling and cluster detection techniques were employed to assess variations in suicide rates and identify high-risk clusters, alongside examining associations with various risk factors. The findings indicate diverse global and regional age-standardized suicide trends, with overall rates decreasing from an average of 12.97 deaths per 100,000 population in 2000 to 9.93 deaths per 100,000 in 2019. Significant regional variations were noted, particularly in Europe, Asia, and Africa, where high-risk clusters were identified. Additionally, age and sex-specific trends revealed consistently higher rates among males, although these rates have been declining over time. Spatial maps illustrated hotspots of elevated suicide rates, which can inform targeted intervention strategies. Risk factor analysis further revealed associations with socioeconomic and health indicators. The results underscore the necessity for tailored prevention strategies and highlight the importance of international collaboration and surveillance systems in addressing the complexities of global suicide epidemiology. This study contributes valuable insights into suicide patterns and offers implications for mental health policies worldwide.

**Supplementary Information:**

The online version contains supplementary material available at 10.1038/s41598-025-97064-6.

## Introduction

Suicide is a pressing global public health issue prioritized by the World Health Organization (WHO) in its Comprehensive Mental Health Action Plan^1^. Each year, approximately 800,000 lives are lost to suicide worldwide, making it a significant concern with far-reaching consequences for families, communities, and society as a whole^2^. Notably, suicide’s impact extends beyond the immediate loss of life, affecting population growth, life expectancy, and generational well-being^2^. As a leading cause of age-standardized years of life lost in high-income regions and among the top ten leading causes of death in most parts of the world, its toll on human lives cannot be overstated^1^. Despite its global significance, there exists a stark disparity in suicide rates among countries. While extensive research on suicide has been conducted in high-income countries (HICs), the majority of global suicides and attempts occur in low- and middle-income countries (LMICs)^3^.

Suicide is defined as a death resulting from intentional self-directed injury^4^. It exhibits variations by age, sex, and means of suicide, with multiple underlying risk and protective factors at individual, family, community, and societal levels^5^. Key factors contributing to suicide rates include access to means of suicide^6,7^, mental illness prevalence, and availability of mental healthcare and support services^8,9^. Understanding these factors at an individual level provides valuable insights, but it may not be sufficient for effective primary prevention of suicide, potentially obscuring deeper underlying causes^10^. Moreover, geographic variations in suicide rates add complexity^11^, with health geography literature highlighting the influence of economic, social, and physical environments on area-level health outcomes^12^.

While there exists a considerable body of research on suicide and suicide attempts, analyses of completed suicides at a global scale remain relatively scarce^1,13,14^. Spatiotemporal epidemiology of suicide has predominantly focused on HICs, leaving gaps in understanding the worldwide patterns of suicide mortality^15^. By employing space-time analytical techniques, this research aims to gain a deeper understanding of how suicide trends have evolved over time and across diverse geographical areas. This comprehensive approach will not only shed light on the current state of suicide epidemiology but also provide insights into changes or shifts in patterns in recent years. Additionally, through the analysis of spatiotemporal suicide data, this research will also investigate high-risk clusters and potential risk factors for suicide in countries worldwide, utilizing socioeconomic indicators collected by the World Health Organization (WHO). By refining and updating previous analyses using these space-time methodologies, this study can make significant contributions to the field of global and regional suicide epidemiology, informing more targeted and effective suicide prevention strategies.

The insights generated by this study hold significant implications for global mental health policy, offering invaluable guidance to policymakers and epidemiologists. This research can guide resource allocation and policy formulation geared towards preventing suicide and fostering mental well-being worldwide^16^. By conducting comprehensive spatiotemporal analyses of global suicide rates, we aimed to understand localized risk patterns to inform evidence-based public health policies and interventions. Identifying high-risk clusters and regions with elevated suicide rates enables more efficient resource allocation for tailored prevention initiatives that address specific risk factors. Strengthening mental health services, addressing socioeconomic disparities, and integrating suicide prevention into broader health policies are vital in mitigating suicide’s impact. Insights from this study could serve as a foundation for evidence-based policies, more effectively combating suicide and promoting mental well-being worldwide.

## Methods

### Data source

This study retrospectively analyzed the global suicide situation and risk factors using data collected from the Global Health Observatory data repository (GHO), WHO’s repository for health-related statistics of its member countries. Global suicide rate estimates were at the country level, yearly, during 2000–2019 across 183 countries in 5 regions: Africa, America, Asia, Europe, and Oceania^17,18^. This data represented suicide rates. The world geographic country boundaries were obtained from the GEO package file in the Global Administrative Region Database (GADM), which is a high-resolution territorial database containing provincial to subdistrict data for all countries across the world. The data files for analysis were created by combining the R version 4.2.1 and Python version 3.8 programming languages. More details about risk factor data can be found in the supplementary document S1, which outlines the data collection and risk factor selection procedures.

### Spatiotemporal bayesian modelling and cluster detection

While variations in suicide risk between national and subnational units may be influenced by unobserved confounders, extensive research has identified a relationship between suicide rates and socio-economic factors and cultural beliefs. However, it is important to acknowledge that apparent spatial patterns of suicide risk are often assessed through standardized mortality rates (SMRs). Conventional estimators treat each area independently, ignoring regional context and neighboring associations^19^. In contrast, multilevel or Bayesian methods use information from all areas, leading to more stable estimates, especially for small areas or space-time combinations^20^.

This study applied Bayesian spatiotemporal modeling to investigate global suicide rate patterns over time and across geographic regions. By integrating spatial and temporal dependencies, this approach stabilizes SMR estimates by leveraging information from neighboring areas and regional trends. The model also facilitates a more detailed examination of the relationship between age-standardized suicide mortality rates and multiple risk factors, providing a deeper understanding of suicide epidemiology.

Age-standardized mortality rates are widely used to assess mortality while accounting for differences in population age structures, which can vary significantly between countries and regions^21,22^. The WHO Global Health Estimates on suicide rates incorporate data from multiple sources to provide the most reliable estimates possible^23^. Standardizing mortality rates per 100,000 individuals using WHO’s standard population distribution enables meaningful comparisons across populations with differing age structures^21^. This metric is essential for tracking suicide trends, identifying health disparities, and evaluating the effectiveness of public health interventions. The findings from this analysis can offer insights into the complex interactions between risk factors and suicide rates, potentially informing evidence-based suicide prevention strategies and shaping global mental health policies.

The age standardized mortality rates in this study were assumed to follow a Log-Normal likelihood. So, let $\:{y}_{it}$ be the age standardized mortality rate for location $\:i$ and time $\:t$. A common way to analyze a log-normal variable $\:{y}_{it}$ is to log-transform $\:\left({z}_{it}=log\left({y}_{it}\right)\right)$ so that it follows a normal distribution with expected value $\:{\mu\:}_{z,t}$, t and standard deviation $\:{\sigma\:}_{z}$. The geometric mean of $\:{y}_{it}$ is then found as $\:\text{e}\text{x}\text{p}\left({{\upmu\:}}_{\text{z},\text{i}\text{t}}\right)$ while the expected value of $\:{y}_{it}$ (the arithmetic mean) is found as $\:{{\upmu\:}}_{y,it}=exp\left({{\upmu\:}}_{z,it}+\frac{{{\upsigma\:}}_{z}^{2}}{2}\right)$. The expected value $\:{{\upmu\:}}_{y,it}$ depends on several predictors, regression analysis is often based on the log-transformed outcome. This can be expressed as ${z_{it}}\sim Normal({\mu _{z,it}},\sigma _{{z,it}}^{2})$ and ${\mu _{z,it}}={\beta _0}+\sum\nolimits_{m}^{{}} {{\beta _{it}}} X_{{it}}^{{}}+{\eta _{it}}$ where ${\beta _0}$is the overall intercept, ${\beta _{it}}$ is the spatiotemporal association coefficients, ${X_{it}}$is risk factors, and $\eta _{{it}}$ is the space-time random effects.

The risk factor association was modeled on the multiplicative scale. Conditioned on the random effect terms and other fixed effects constant, the interpretation was that the overall geometric mean of age standardized suicide mortality rate for risk factor $\:m$ was expected to change by $\:100$(exp(*β*_*it*_-1) percent as the risk factor increases one unit. To specify the space-time Bayesian models, three types of spatial random effects, three types of temporal effects, and four types of spatiotemporal interaction terms were considered. Consequently, a total of 36 combinations were assessed to determine the optimal spatiotemporal mixed structure for suicide modeling. More details of model specifications can be found in the supplementary document S1.1.

Spatiotemporal cluster detection is an important tool in public health and many other areas of application. Spatiotemporal clustering is an extension of spatial clustering in which the time dimension is introduced into spatial data^24,25^. In spatiotemporal clustering, the objects are grouped as per their spatial and temporal similarity^26^. Thus, in this study, a suicide cluster or anomaly can be identified using the exceedance probabilities as $p({\mu _{z,it}}>{q_t})$ where $\:{q}_{t}$ is the median age standardized mortality rate at year *t*. Then, a hotspot was defined as a location with$p({\mu _{z,it}}>{q_t})$ greater than a cut-off point, i.e. $p({\mu _{z,it}}>{q_t})>1 - \alpha$ where $\:\alpha\:$was the pre-specified level of significance^27,28^. In the study, the baseline threshold was set as the median of suicide rates among the countries worldwide at the level of significance = 0.05. The resulting hotspots were defined based on the probability of exceedance. That is the result of cluster detection reveals countries that exhibit above-average suicide rates, referred to as hotspots, during the period from 2000 to 2019.

### Model assessment metrics and two-step model selection

After constructing several model specifications to account for the spatiotemporal variation in spatial health data, a model selection process was employed to identify the simplest model that best fit the observed data. This approach ensured robust and reliable predictions of suicide incidence and its associations with potential risk factors^29^. To achieve the optimal spatiotemporal mixed structure, a top-down two-step procedure was applied^29,30^. In the first step, a set of risk factors was identified to define the mortality association structure. Once the optimal risk factor structure was determined, the second step involved refining the model by testing various combinations of random effects. The best-fitting model was then selected based on multiple evaluation criteria.

For the fixed-effect selection, spatial and temporal risk factors were chosen based on a systematic review of suicide studies^11^. These included health and socioeconomic indicators sourced from the Global Health Observatory data repository. The primary objective of the model selection process was to identify the simplest, most interpretable model that optimally fit the observed data. To further enhance clarity and reduce redundancy, correlation analysis was performed on the potential risk factors. The strength of relationships between variable pairs was assessed, and to prevent multicollinearity, only one risk factor was retained from highly correlated pairs, based on its strongest correlation with the suicide data. Additional details on risk factor selection are provided in supplement S1.2.

In selecting the random effects, we compared different combinations of space-time fixed and random effects using standard model fit metrics commonly employed in hierarchical modeling^31,32^. These included the Deviance Information Criterion (DIC), Watanabe-Akaike Information Criterion (WAIC), bias, root mean squared error (RMSE), conditional predictive ordinate (CPO), computation time, and correlation coefficient. The analyses and visualization of the results were performed using R and Matplotlib in Python. The selected model aimed to strike a balance between statistical and epidemiological considerations, effectively translating the findings to inform practical plans for suicide prevention. By applying a Bayesian framework, uncertainty quantification was considered, and coefficients were deemed significant when their exceedance probability exceeded the pre-specified level of 0.05. More details on model comparison and evaluation metrics are presented in supplementary document S1.3.

## Results

### Global and regional age-standardized suicide trends

**Fig. 1:**
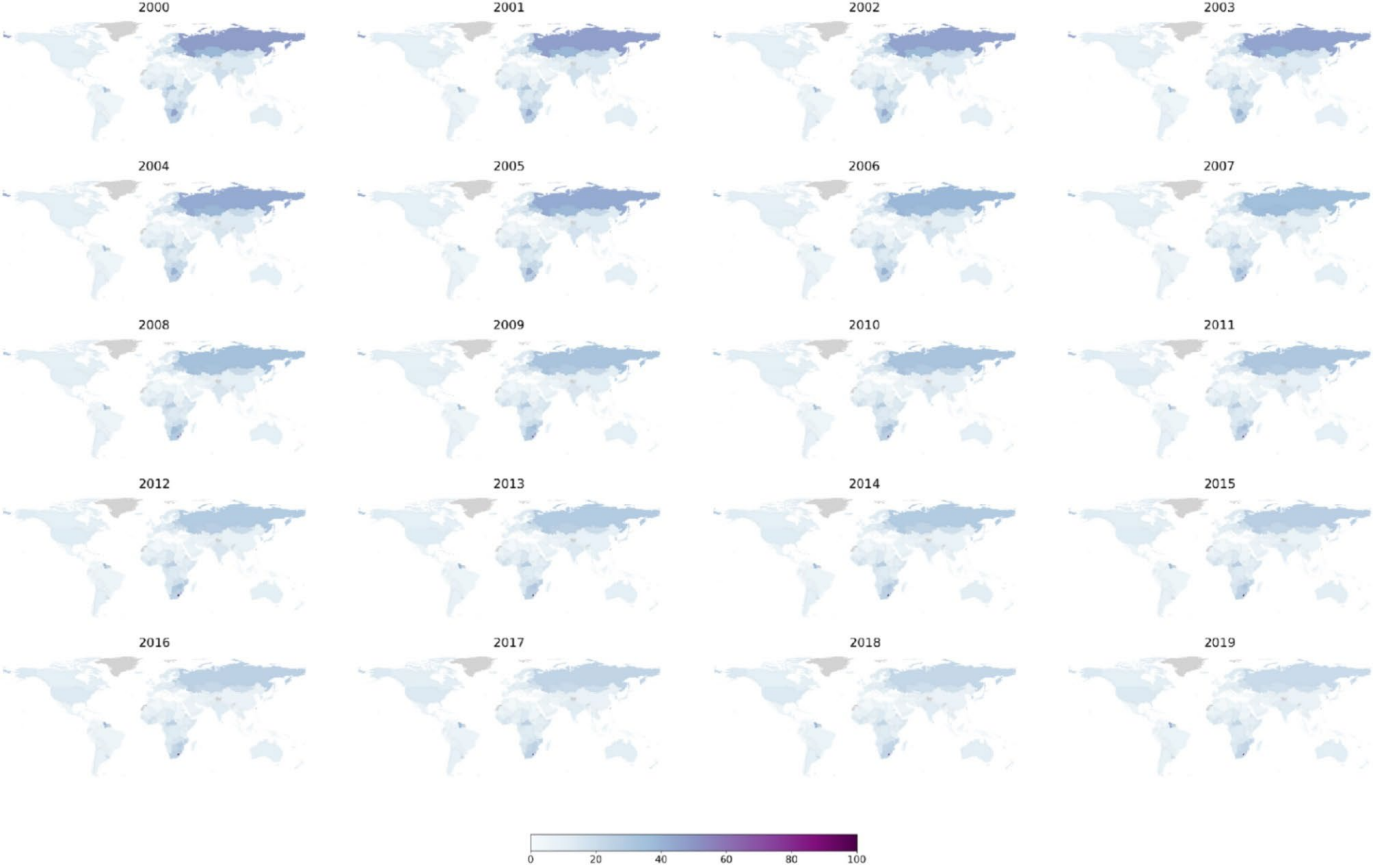
Global maps of country level age standardized suicide rates per 100,000 population 2000–2019, generated using RStudio version 2022.07.0 + 548 (available at https://posit.co/products/open-source/rstudio/).

**Fig. 2:**
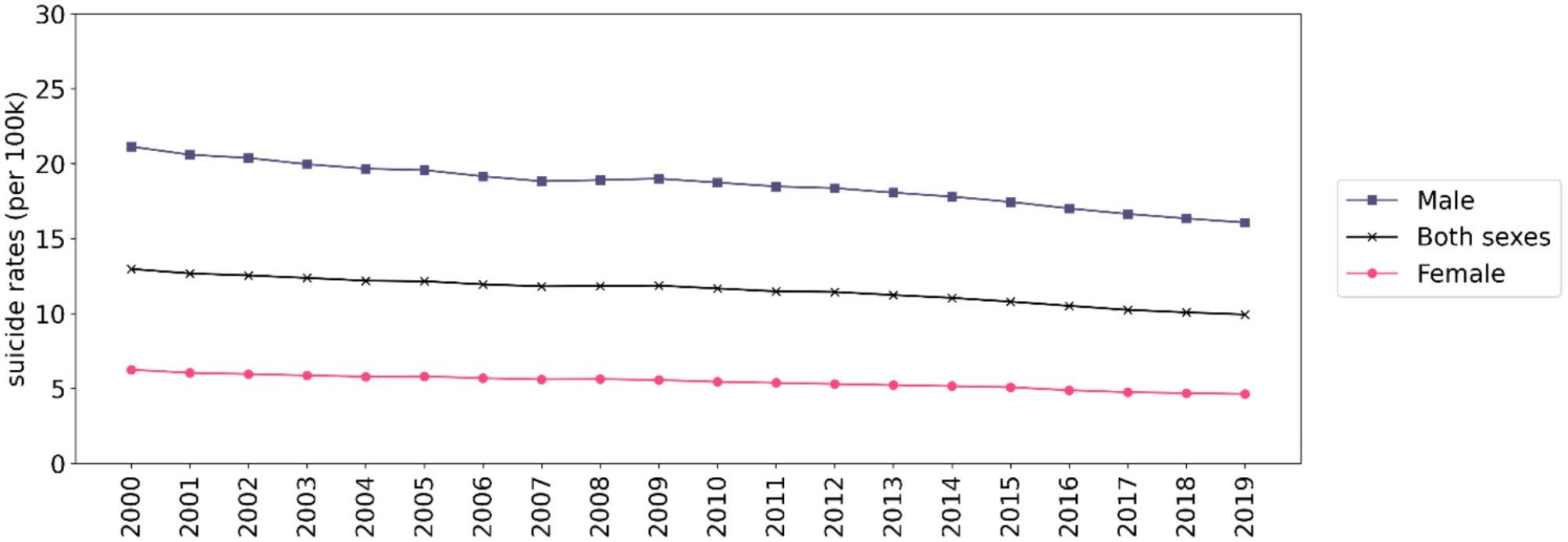
Plot of global age-standardized suicide rates by sex (2000–2019) per 100,000 population.

The descriptive analysis encompassed data from 183 countries across 5 regions: Asia (47 countries), Africa (54 countries), Americas (33 countries), Europe (39 countries), and Oceania (10 countries). The study visualized both age-standardized suicide rates during 2000–2019 (per 100,000 population) and age-specific suicide rates in 2019 (per 100,000 population). Globally, the average age-standardized suicide rate in 2000 was 12.97 deaths per 100,000 population, experiencing a slight and steady decrease of 23.44% to 9.93 deaths per 100,000 population in 2019. Figure 1 demonstrates the overall downward trend in country-level age-standardized suicide rates from 2000 to 2019. For additional yearly global maps for each sex from 2000 to 2019, please refer to the supplementary document S2. In 2000, Africa, Europe, and Oceania exhibited higher average age-standardized suicide rates, at approximately 16 deaths per 100,000 population, compared to Asia and the Americas, which reported average rates of 8.5 deaths per 100,000 population in the same year. Furthermore, Fig. 2 displays age-standardized suicide rates by sex in 2000, 2005, 2010, 2015 and 2019 per 100,000 population, consistently showing higher rates for males compared to females over time.

**Fig. 3:**
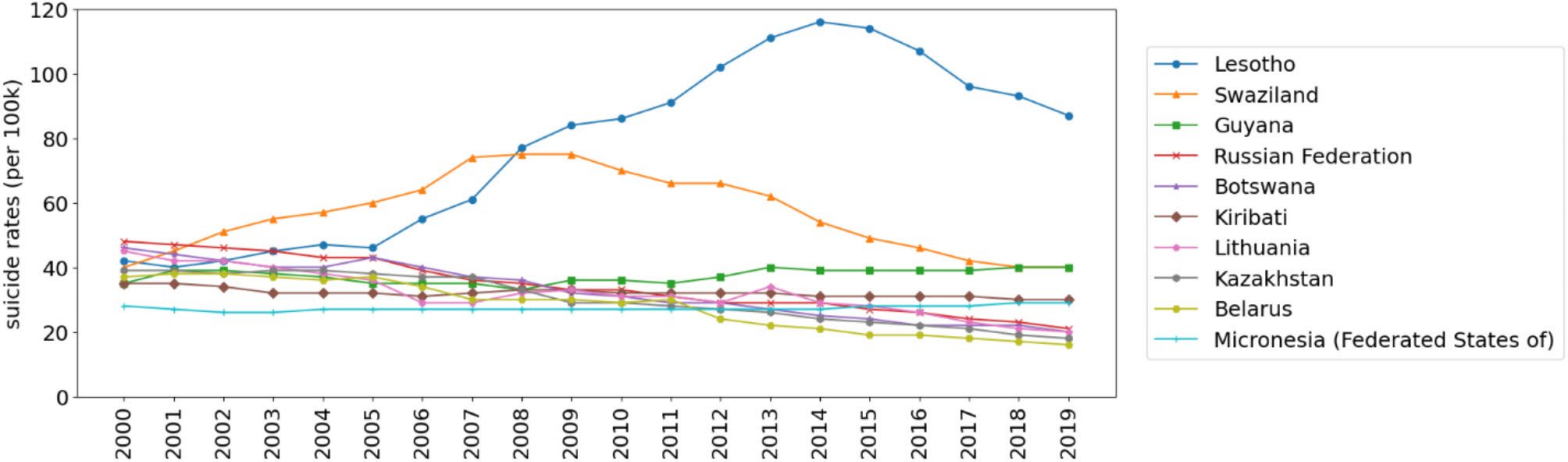
Plot of top ten countries with highest average global age-standardized suicide rates (2000–2019) per 100,000 population.

Figure 3 illustrates the trends in age-standardized suicide rates for the 10 countries with the highest mean rates from 2000 to 2019; these were 3 countries in Africa (Lesotho, Swaziland, and Botswana), 3 countries in Europe (Russian Federation, Lithuania, Belarus), 2 countries in Oceania (Kiribati and Micronesia (Federated States of)), 1 country in Asia (Kazakhstan), and 1 country in the Americas (Guyana). In 2000 the highest rates were observed in the Russian Federation, Botswana, Lithuania, Lesotho, and Swaziland. By 2005, the age-standardized suicide rates in Swaziland rose to as high as 60 deaths per 100,000 population, becoming the highest among all countries. In the same year, Lesotho also experienced an increase in rates to 46 deaths per 100,000 population.

The trend continued in 2010, with Lesotho and Swaziland’s age-standardized suicide rates continuously increasing to 86 and 70 deaths per 100,000 respectively. However, in 2015, while Lesotho’s rates increased to 114 deaths per 100,000, other countries, including Swaziland, showed a decrease. By 2019, the age-standardized suicide rates had decreased in every country. Despite only a few countries having high rates of suicide mortality, they significantly influenced the regional averages. Figures in the supplementary document S3 indicates that most regions’ suicide rates remained relatively stable throughout the study period, except for Europe, where age-standardized suicide rates decreased by 37.15% from 16.26 deaths per 100,000 in 2000 to 10.22 deaths per 100,000 in 2019.

### Sex- and age-specific suicide trends

**Fig. 4:**
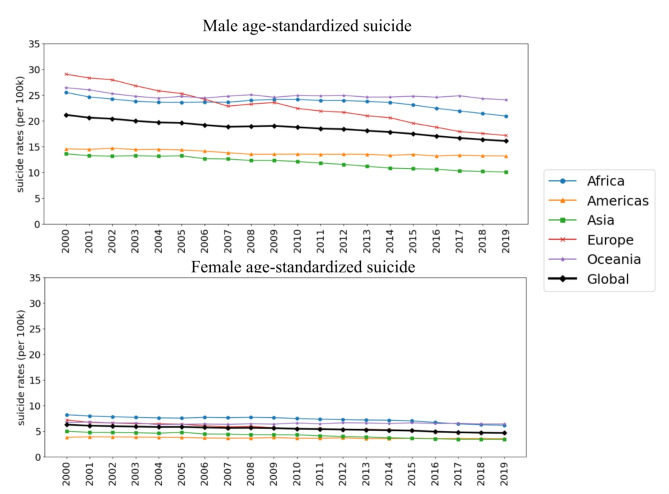
The plot displays global male and female age-standardized suicide rates by region from 2000 to 2019, per 100,000 population.

**Fig. 5:**
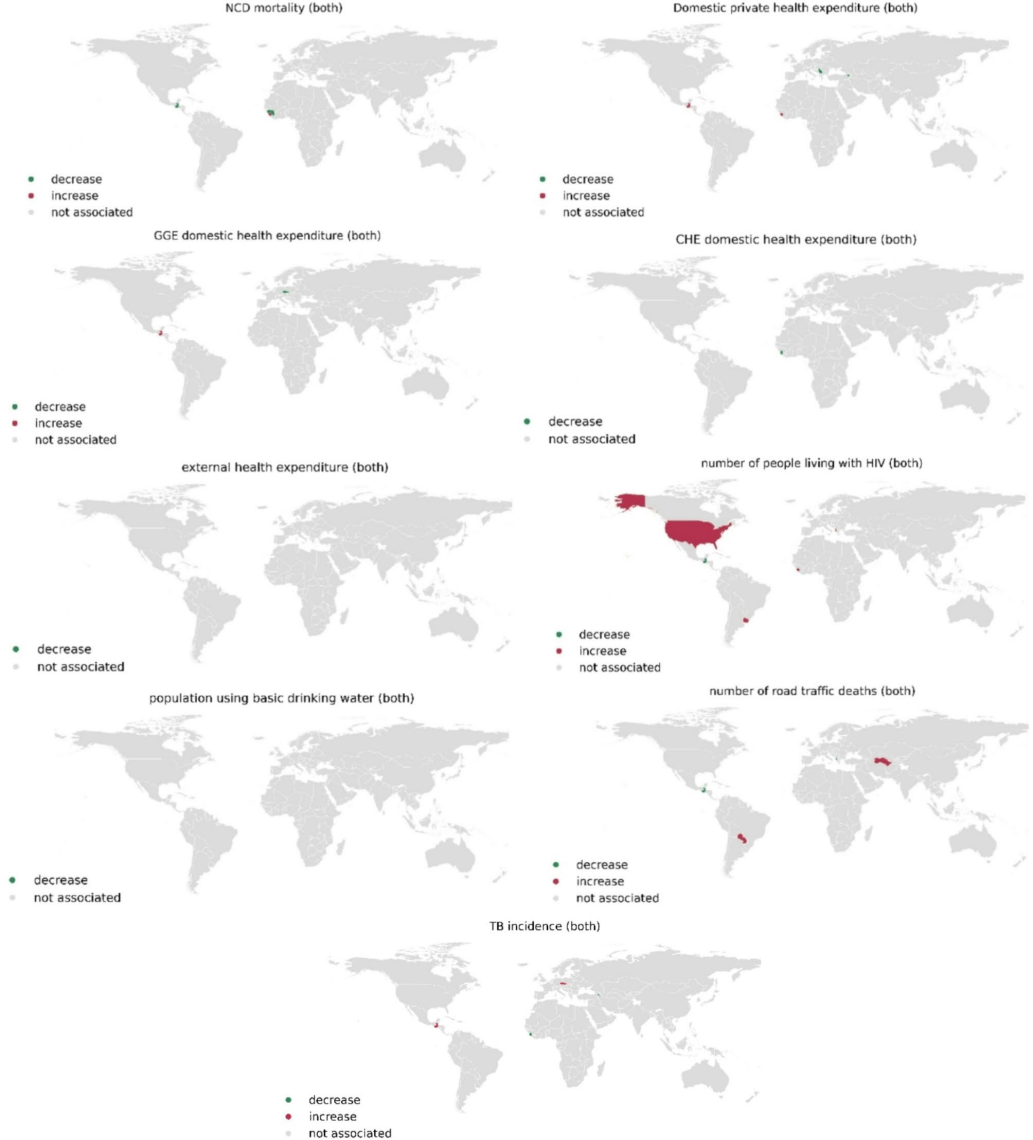
Global maps illustrating the overall association between risk factors and age-standardized suicide rates for both sexes combined, generated using RStudio version 2022.07.0 + 548 (available at https://posit.co/products/open-source/rstudio/).

During the study period, there was a notable difference in age-standardized suicide rates between males and females. The global average age-standardized suicide rates were consistently higher among males compared to females throughout the entire study period (Fig. 4). However, both male and female age-standardized suicide rates showed a decreasing trend over time. Male age-standardized suicide rates decreased by 23.98%, from 21.14 deaths per 100,000 in 2000 to 16.07 deaths per 100,000 in 2019. Similarly, female age-standardized suicide rates decreased by 25.80%, from 6.24 deaths per 100,000 in 2000 to 4.63 deaths per 100,000 in 2019. Figure 5 displays the regional trends in age-standardized suicide rates for both sexes, showing that Africa and Europe had the highest rates, consistent with regional trends. Global maps of country-level age-standardized and age-specific suicide rates are presented in figures in the supplementary document S3.

Additionally, geographical plots for global age-specific suicide rates in 2019 revealed varying suicide rates across different age groups (Figure S8). The data covered 8 age groups: 15–24 years, 25–34 years, 35–44 years, 45–54 years, 55–64 years, 65–74 years, 75–84 years, and 85 years old and above. When mapped globally, most countries exhibited similar suicide rates in the 15–24-year age group up to the 45–54-year age group. However, differences in suicide rates became more prominent starting from the 55–64-year age group, and the rates were highest in the 85 years and older age group. Note that the country-level age-specific suicide rates were available for the year 2019 only. Global maps of male age-specific suicide rates in 2019 and global female age-specific suicide rates in 2019 per 100,000 population are depicted in Figure S8. These maps reveal significant differences both between sexes and among the age groups. In 2019, male suicide rates were consistently higher than female suicide rates across all age groups, as also evidenced in the age and sex trends.

### Space-time cluster detection and association with risk factors

#### Hotspot cluster detection

The global maps of country-level age-standardized suicide hotspots during 2000–2019 are shown in figures in the supplementary document S4. while the hotspot maps of both sexes combined are depicted in Figure S16 in the supplementary document S4. In the visualizations, hotspots are red and non-hotspot areas are blue. Among the identified hotspots are European countries including Russia, France and Poland, Asian countries including Japan, Mongolia, Kazakhstan, and India, as well as many countries in southern and central Africa. The countries identified as hotspots each year were mostly the same from 2000 to 2019. When female and male suicide data were compared spatially, India and China consistently emerged as hotspots for female suicide from 2000 to 2019 but not for males. Since 2003, the United States had a higher-than-average male suicide rate (hotspot), and from 2016 to 2019, females in the United States and Canada also began to appear as a hotspot. Furthermore, Australia was a hotspot for female suicide rate only in 2017.

#### Spatial association of suicide with risk factors

Various model combinations of space-time fixed and random effects were compared by assessing their fit metrics, however the primary measure was based on the widely applicable information criterion to identify the best model combinations. The best random effects for data with combined sexes and for females included the convolution model for spatial component, random walk order 2 for temporality and the space-time interaction type one, whereas the best random effects for males were the Besag model with random walk order 2 for temporality and the space-time interaction type one. The full results of model fitting and comparison are provided in the supplementary document S5. Ten potential risk factors were selected for analysis based on their correlation coefficients. To avoid multicollinearity, pairwise comparisons were conducted, and from each highly correlated pair, only the risk factor exhibiting the stronger correlation with suicide rate was chosen. The selected risk factors included age-standardized NCD mortality rate (per 100,000 population), current health expenditure (CHE) as a percentage of gross domestic product (GDP), domestic general government health expenditure (GGHE-D) as a percentage of current health expenditure (CHE), domestic general government health expenditure (GGHE-D) as a percentage of general government expenditure (GGE) (%), domestic private health expenditure (PVT-D) per capita in US dollar, external health expenditure (EXT) per capita in US dollar, estimated number of people (all ages) living with HIV, estimates number of road traffic deaths, population using at least basic drinking-water services, and incidence of tuberculosis (per 100,000 population per year).

Figure 5 presents global maps depicting the overall associations between risk factors and age-standardized suicide rates for both sexes combined. The analysis findings revealed that 9 out of 10 studied risk factors exhibited a strong association with suicide rates using 95% credible interval. The only exception was current health expenditure as a percentage of gross domestic product. Moreover, it was observed that the association between risk factors and suicide risk was observed primarily in a limited number of countries, mostly in the Americas region. Several countries, including Guatemala, Sierra Leone, and Barbados, consistently displayed associations with multiple risk factors. Domestic private health expenditure per capita was found to have a significant association with suicide risk in 7 out of 181 countries including Antigua & Barbuda, Armenia, Greece, Russian Federation, Saint Lucia, Sao Tome and Principe, and Senegal. In some of the associated countries, an increase in domestic private health expenditure per capita was correlated with an increase in suicide risk, while in others, it was associated with a decrease in suicide risk for both male and female populations.

In 9 out of 181 countries, predominantly in the Americas, including Albania, Antigua & Barbuda, Barbados, Ghana, Greece, Saint Lucia, Senegal, U.K., and United Republic of Tanzania, a significant correlation was discovered between the estimated number of people (all ages) living with HIV and the risk of suicide. In some countries, an increase in the estimated number of people living with HIV was associated with an increase in suicide risk, indicated by the color red, including Albania, Antigua & Barbuda, Grenada, Sao Tome and Principe, Sierra Leone, United States of America, and Uruguay, while in others, it was associated with a decrease in suicide risk, indicated by the color green, including Barbados and Guatemala. A significant association was observed in 8 out of 181 countries including Antigua & Barbuda, Armenia, Barbados, Czech Republic, Grenada, Guatemala, Sao Tome and Principe, and Sierra Leone, between the incidence of tuberculosis (per 100,000 population per year) and suicide risk. The analysis indicated a significant association between the age-standardized non-communicable disease (NCD) mortality rate (per 100,000 population) and suicide risk in a total of 7 out of 181 countries, mainly in the Americas including Antigua & Barbuda, Barbados, Grenada, Guatemala, and in Africa including Guinea, Sao Tome and Principe, and Sierra Leone. In most of the countries where an association was observed, an increase in the NCD mortality rate was found to be linked to an increased risk of suicide, as indicated by the color red. These results were consistent for both male and female populations. More details of spatiotemporal association analysis and plots for each factor can be found in supplementary document S6.

## Discussion

Between 2000 and 2019, several countries in Europe, Asia, and Africa consistently emerged as hotspots for both male and female suicide rates; however, significant associations with risk factors were only found in a few countries. Notably, there were changes in suicide rates over time, particularly in Europe. These changes could be influenced by various factors, including economic fluctuations, shifts in societal norms and values, access to mental health care, and public health initiatives aimed at suicide prevention. For instance, economic crises in Europe have been linked to increases in suicide rates^33^, suggesting that economic stability plays a crucial role in suicide prevention. Additionally, societal attitudes towards mental health, increased awareness, and availability of mental health services may also contribute to these variations.

The finding that associations between risk factors and suicide rates vary spatially and temporally highlights the complexity of factors contributing to suicide. For example, while an increase in health expenditure was generally associated with a decrease in suicide risk, indicating that better access to healthcare can reduce suicide rates, the reverse association in some contexts might suggest that higher suicide rates lead to increased awareness and investment in mental health services. This bidirectional relationship underscores the need for a nuanced understanding of how different factors influence suicide rates and the importance of continuous monitoring and evaluation of suicide prevention strategies.

An association between suicide risk and health expenditure was observed in various countries, particularly in the Americas and Africa. In most of these countries, an increase in domestic general government health expenditure as a percentage of current health expenditure and external health expenditure per capita was associated with a decrease in suicide risk. This could be because healthcare costs cover both physical and mental illnesses, and more patients receiving treatment can prevent serious diseases or suicidal tendencies. One study also suggested that a strong social support system, such as family networks and welfare expenditures, may protect against suicide by providing support and ensuring compliance with medical or psychiatric treatment^34^. It is essential to support government health expenditures to aid in suicide prevention.

The relationship between the number of road traffic deaths and suicide estimates was inconsistent. A previous study found no conclusive evidence of an association between car accidents and suicide^35^. Additionally, there was limited information on suicides and natural deaths in road traffic accidents, particularly pedestrian suicides. Driver suicides were more common among males aged 25–34 years and were often associated with factors such as previous suicide attempts, mental illness history, and alcohol consumption. Conversely, natural driver deaths mostly occurred among males aged 50 to 70 years, primarily due to cardiovascular disease, with minimal property damage. Unfortunately, data on pedestrian suicides or natural deaths were often lacking or insufficiently detailed in national and international road safety or other databases, making it difficult to fully understand the relationship between road traffic fatalities and suicide^36^.

Male suicides were more prevalent than female suicides in most countries. This might be because suicide methods chosen by men are often more violent and less likely to be interrupted by intervention^37^. Access to means is also a crucial factor, particularly in the United States, where six out of ten gun owners are men^38^ and firearms account for 46% of all suicides^39^. Additionally, differences in emotional expression and communication between sexes play a role, as women tend to be more willing to share their problems than men^40^. Other contributing factors to male suicides include occupational stress and alcohol abuse^41^. China had a higher-than-average suicide rate for females only, with no significant association with the studied risk factors. The higher female suicide rates in China can be attributed to traditional expectations for women to be subservient to men, and sex-based discrimination^42^.

The findings of our study reveal mixed directional associations between the examined risk factors and suicide rates across countries. In most cases, the associations were non-significant, while among the significant findings, some demonstrated an inverse relationship, and others showed a direct one. This variability is consistent with findings from other studies^11,43^, suggesting that the relationship between area-level socio-economic characteristics and suicide rates may differ depending on contextual and cultural factors. Additionally, the relationship may be mediated by other contextual characteristics. For instance, an English study highlighted that social fragmentation in an area could act as a mediating pathway between area-level deprivation and suicidal behavior^44^. Similarly, a Spanish study demonstrated that the association between area-level deprivation and higher suicide rates was influenced by city size^45^. These examples underscore how broader contextual factors can shape the direction and strength of these associations.

While this research has yielded valuable insights in global suicide epidemiology, it is important to acknowledge its inherent limitations. The study relied on retrospective analysis of suicide data, which may be subject to underreporting and biases. Furthermore, the analysis focused on global suicide rate estimates at the country level, which might mask finer spatial variations within specific sub-regions or populations. In addition, the study examined the association of selected risk factors with suicide rates, but other unexplored factors may contribute to suicide risk. Each of the risk factor datasets have their own limitations and biases which could not be accounted for in the analysis.

Nonetheless, this study’s findings have important implications for global health policy. Understanding the complex interplay of risk factors contributing to suicide on a global scale can guide the development of more effective and targeted suicide prevention strategies. By identifying regions and populations with higher suicide rates and their associated risk factors, policymakers and public health organizations can allocate resources and implement interventions where they are most needed. This research underscores the global significance of suicide prevention efforts and provides evidence to support the integration of mental health promotion into broader global health agendas.

Having a comprehensive understanding of global suicide trends, geographic distribution, and associated risk factors is important for informing effective suicide prevention strategies on a global scale. By analyzing trends and identifying high-risk regions and populations, policymakers and public health organizations can strategically allocate resources and implement targeted interventions where they are most needed. This analysis serves as a crucial foundation for the development of impactful regional and global strategies and guidelines aimed at reducing suicide rates and promoting mental health worldwide. It underscores the urgent need for the integration of mental health promotion into broader global health agendas, emphasizing the global significance of suicide prevention efforts.

## Conclusion

This study offers a comprehensive understanding of country-level global suicide patterns, underlining the urgency of grasping this widespread public health concern. Through diverse data analysis, we have uncovered insights that highlight suicide’s multifaceted nature and the imperative for tailored prevention strategies. While economic and sociodemographic factors contribute, our research underscores the need to delve deeper into the global suicide landscape, particularly its spatiotemporal dimensions. The variation in suicide rates across regions underscores the need for targeted interventions adapted to specific contexts. Collaborative efforts among international organizations, policymakers, and researchers are essential to addressing this global challenge. By identifying commonalities and differences in suicide rates and risk factors, our findings lay the groundwork for evidence-based strategies that can effectively reduce global suicide rates. This collaborative approach resonates with the broader objective of advancing global mental health policies, contributing to collective efforts to mitigate the impact of suicide on societies worldwide.

## Electronic supplementary material

Below is the link to the electronic supplementary material.

Supplementary Material 1

## Data Availability

The data supporting the findings of this study are publicly accessible from the Global Health Observatory (GHO) data repository, available at https://www.who.int/data/gho.
