# Supplementary material for: Global spatiotemporal analysis of suicide epidemiology and risk factor associations from 2000 to 2019 using Bayesian space time hierarchical modeling
Source: Sci Rep. 2025 Apr 14;15:12785. doi: 10.1038/s41598-025-97064-6 (PMC11997172; doi:10.1038/s41598-025-97064-6)
Supplement: Supplementary file 1 — Supplementary Material 1 [file 41598_2025_97064_MOESM1_ESM.docx]

**Global spatiotemporal analysis of suicide epidemiology and risk factor associations from 2000 to 2019 using Bayesian space time hierarchical modeling**

**Supplement S1: Spatiotemporal model specification and selection**

*Supplement S1.1: Model specification and cluster detection*

The random effects were defined as a linear combination of space-time terms as .We spatially then structured the random effects by borrowing information across neighboring regions and time periods to incorporate spatiotemporal smoothing. The convolution model was modeled to the spatial random effect as $\gamma_{i}=u_{i}+v_{i}$where$u_{i}$and $v_{i}$were employed to capture spatially correlated and unstructured extra variation in suicide rate distribution. It was important to include both structured and unstructured random effects in a spatial analysis because we did not have strong prior knowledge and unobserved confounders could take several forms. The uncorrelated random effect was modeled by the zero-mean Gaussian distribution whereas the spatial random effect was described by the intrinsic conditional autoregressive model (ICAR) proposed by Besag et al. [1]. That is, conditionally, $u_{i}|u_{-i}\sim N\left(\bar{u}_{\Omega_{i}},\sigma_{u}^{2}/n_{i}\right)$where $u_{-i}$is the vector containing the neighboring effect of all except the *i*th area. $\Omega_{i}$, $n_{i}$and $\bar{u}_{i}$are a set of adjacent neighbors, cardinality and the mean of the neighborhood of the *i*th province respectively, and $\sigma_{u}^{2}$ is the spatial component variance.

To model temporal variation, a linearity constraint could be imposed on the differential temporal trend, nonetheless, a dynamic nonparametric formulation might be a better option for the linear predictor as there was no prior specific information for the trend. There are three common forms of temporal random effect. The first one is to model$\delta_{t}$using a Gaussian exchangeable prior as$\delta_{t}\sim Gaussian(0,\sigma_{\delta}^{2})$. The other two are the random walk (RW) priors of order 1 (RW1) and 2 (RW2) which can be expressed as $\delta_{t}^{RW1}|\delta_{t-1}^{RW1}\sim Gaussian(\delta_{t-1}^{RW1},\sigma_{\delta}^{2})$for RW1 and $\delta_{t}^{RW2}|\delta_{t-1}^{RW2},\delta_{t-2}^{RW2}\sim Gaussian(2\delta_{t-1}^{RW2}+\delta_{t-2}^{RW2},\sigma_{\delta}^{2})$for RW2. $\sigma_{\lambda}^{2}$is the variance of the temporal random effect. The description of depends on the spatial and temporal random effects assumed to interact in the model. There are different types of interactions proposed in Bayesian disease mapping literature [2]. However we adopted four commonly used interaction forms [3] in this study.

For type I interaction ($\theta_{1,it}$), the random effect was assumed to be interaction between the non-spatial, $v_{i}$, and exchangeable Gaussian temporal, $\delta_{t}$, terms. According to Knorr-Held notation [2], the structure matrix$R_{\theta}$for the prior ofcan be expressed as the Kronecker product of the interacting random effects. For the first type of interaction, the structure matrix can be written assince both$v_{i}$and$\delta_{t}$do not have a specific spatiotemporal structure. Note that ***I*** here is the identity matrix. For the type II interaction (), the interaction term combines the non-spatial with structured temporal random effects. Then the structure matrix can be described aswhere$R_{v}=I$andis based the neighborhood structure specified by the order of random walk model. Socan be formulated from the assumption of an autoregressive structure on the time component, which is independent from the ones of the other locations. The matrixthen has a rank of *I*(*T* − 1) for a first-order and *I*(*T* − 2) for a second-order random walk model. For the last type of interaction,combines the unstructured temporal effect$\lambda_{t}$and the spatially structured effect$u_{i}$. The structure matrix hence can be written aswhere$R_{u}=I$and$R_{\delta}$is described through the intrinsic conditional autoregressive model. This results in the interaction with a spatial structure independent from the other time points and the structure matrixhas a rank of *T*(*I* − 1). Therefore, a total of 36 combinations were assessed to determine the optimal spatiotemporal mixed structure for suicide modeling.

*Supplement S1.2: Suicide risk factor selection*

In this study, we evaluated 37 potential risk factors for their association with suicide rates, using data from the Global Health Observatory (GHO), a World Health Organization (WHO) data collection project. These risk factors were carefully chosen to align with prior research on suicide determinants and ensure relevance for the study objectives. The process of selecting and preparing risk factors presented challenges, as the datasets varied in variables, timeframes, and formats, requiring sequential loading and preparation. Despite these challenges, consistent preparation steps were applied to both the risk factor and suicide datasets to ensure compatibility for spatiotemporal modeling.

The selection of risk factors was guided by previous studies and systematic reviews on suicide-related research. Socioeconomic and area-level variables, widely acknowledged as significant contributors to suicidal behavior, were prioritized. For instance, a systematic review across 14 European countries between 2005 and 2015 found strong associations between area-level socioeconomic disadvantage and suicidal behavior or self-harm [4]. This study established a strong foundation for prioritizing economic and social factors as primary criteria for analysis. Additionally, health-related factors were incorporated, as some studies highlighted their role in suicide mortality. For example, a study on people living with HIV/AIDS (PLWH) revealed that the geographic distribution of HIV prevalence was inconsistent with suicide mortality, emphasizing the need to consider multiple dimensions [5]. Thus, this study combined risk factors from economic, physical health, and social dimensions to comprehensively examine their associations with suicide mortality across countries. The selection process and risk factor categories are detailed in Figures S1 and S2.

The criteria for risk factor selection were based on their relevance, availability, and temporal consistency. The selected risk factors had to align with categories identified in prior systematic reviews, be publicly available as WHO indicators, and have annual data from 2000 to 2019 to ensure temporal consistency with the suicide dataset. Due to the unavailability of specific risk factor data for two countries, the final dataset included 181 countries instead of the original 183. The overall selection process, which ensured the inclusion of high-quality and relevant variables, is illustrated in Figure S2.


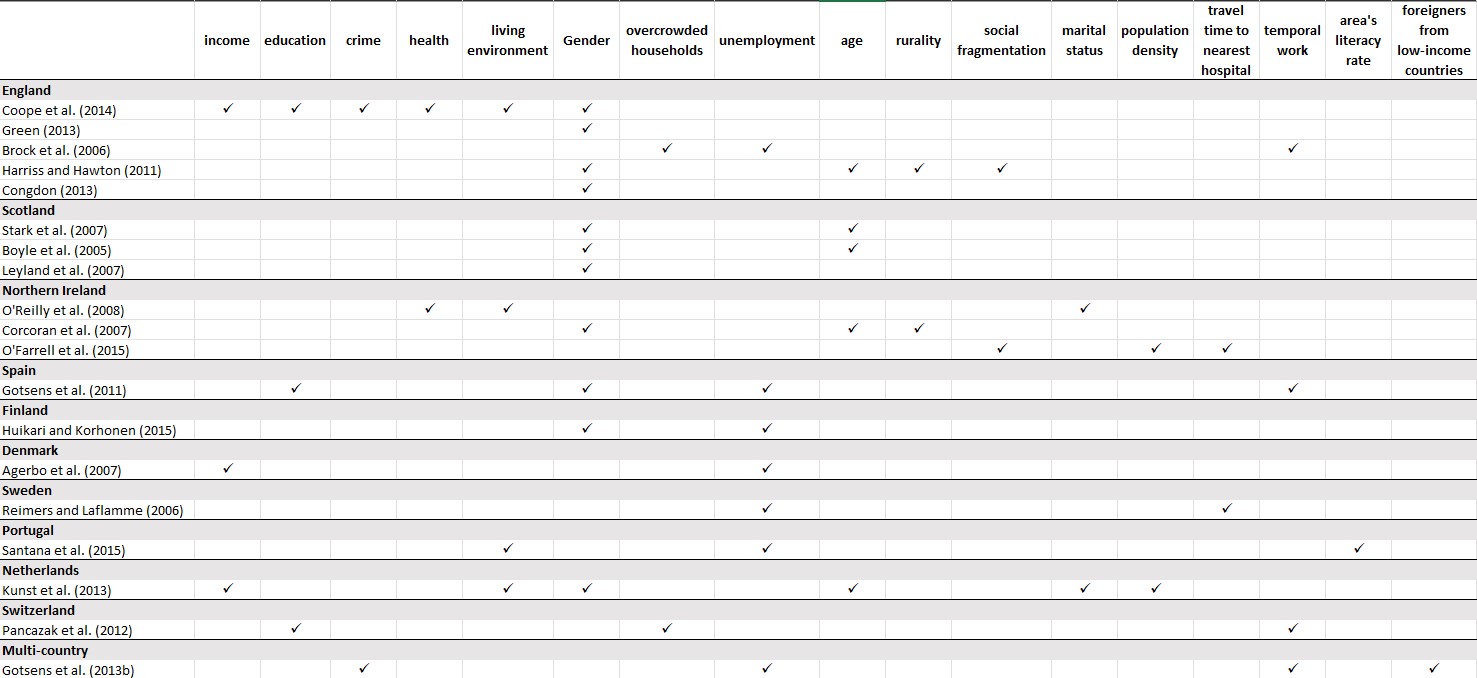


**Figure S1** Potential risk factors for suicide derived from a systematic review of studies conducted in Europe [4].


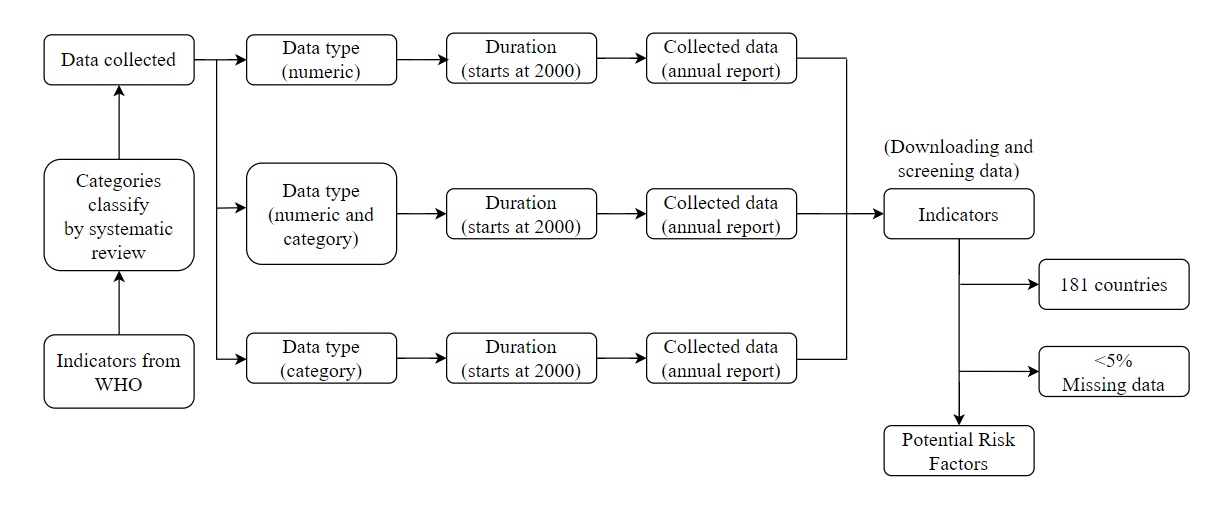


**Figure S2** Criteria for the selection of potential global risk factors.

In model selection, the primary objective is to identify the simplest model that optimally fits the observed data while maintaining interpretability. To reduce redundancy and enhance model clarity, correlation analysis was performed on the 37 potential risk factors. The Pearson’s correlation coefficient was calculated to measure the strength of the relationships between pairs of variables, with coefficients ranging from -1 to 1. Pairs of variables with correlation coefficients exceeding 0.7 or falling below -0.7, based on Ratner’s criteria [6], were flagged for further evaluation. For each highly correlated pair, the correlation of each variable with the target variable (age-standardized suicide rate) was computed. The variable exhibiting the stronger correlation with the target variable was retained, while the weaker variable was excluded. This iterative process refined the initial set of 37 variables down to the 10 risk factors most strongly associated with suicide rates. The selected variables—X1, X3, X6, X7, X13, X16, X18, X19, X26, and X33—are detailed in Table S1 (selected variables are highlighted in bold). A summary of the correlation analysis and variable reduction process is presented in Figure S3.


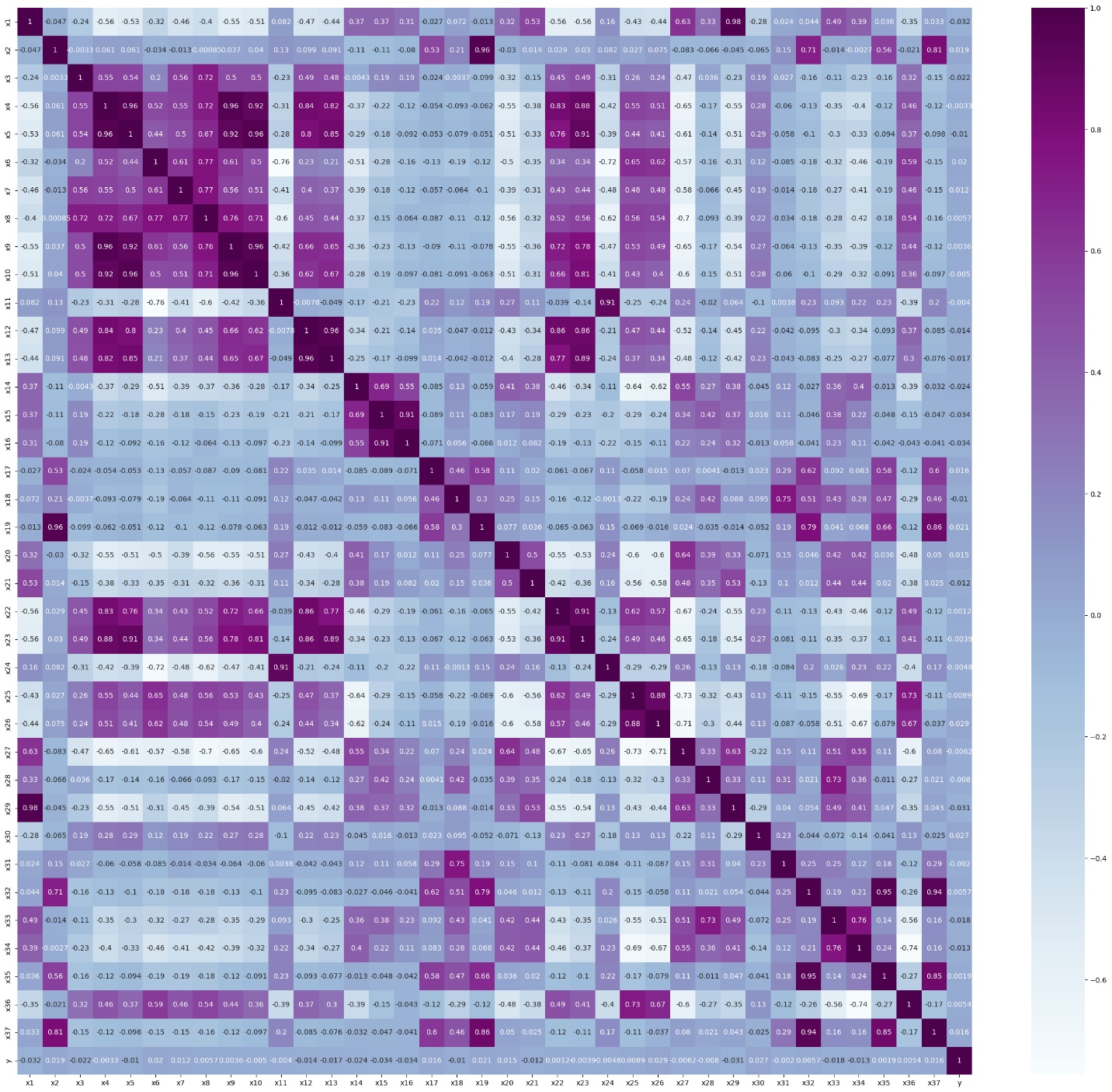


**Figure S3** Correlation coefficient matrix between potential risk factors and target variable.

| **No.** | **Potential Risk Factor name** |
| --- | --- |
| x1 | **Age standardized NCD mortality rate (per 100k population)** |
| x2 | Total NCD Deaths (in thousands) |
| x3 | **Current health expenditure (CHE) as percentage of gross domestic product (GDP) (%)** |
| x4 | Current health expenditure (CHE) per capita in ppp int $ |
| x5 | Current health expenditure (CHE) per capita in US $ |
| **x6** | **Domestic general government health expenditure (GGHE-D) as percentage of current health expenditure (CHE) (%)** |
| **x7** | **Domestic general government health expenditure (GGHE-D) as percentage of general government expenditure (GGE) (%)** |
| x8 | Domestic general government health expenditure (GGHE-D) as percentage of gross domestic product (GDP) (%) |
| x9 | Domestic general government health expenditure (GGHE-D) per capita in ppp int $ |
| x10 | Domestic general government health expenditure (GGHE-D) per capita in US $ |
| x11 | Domestic private health expenditure (PVT-D) as percentage of current health expenditure (CHE) (%) |
| x12 | Domestic private health expenditure (PVT-D) per capita in PPP int $ |
| **x13** | **Domestic private health expenditure (PVT-D) per capita in US $** |
| x14 | External health expenditure (EXT) as percentage of current health expenditure (CHE) (%) |
| x15 | External health expenditure (EXT) per capita in ppp int $ |
| **x16** | **External health expenditure (EXT) per capita in US $** |
| x17 | Estimates of Number of homicides |
| **x18** | **Estimated Number of people (all ages) living with HIV** |
| **x19** | **Estimated number of road traffic deaths** |
| x20 | Estimated road traffic death rate (per 100 000 population) |
| x21 | Mortality rate of unintentional poisoning |
| x22 | Out-of-pocket expenditure (00P) per capita in ppp int $ |
| x23 | Out-of-pocket expenditure (00P) per capita in US $ |
| x24 | Out-of-pocket expenditure as percentage of current health expenditure (CHE) (%) |
| x25 | Population using at least basic sanitation services |
| **x26** | **Population using at least basic drinking-water services (%)** |
| x27 | Premature deaths due to noncommunicable diseases (NCD) as a proportion of all NCD deaths (%) |
| x28 | Prevalence of HIV among adults aged 15 to 49 (%) |
| x29 | Probability (%) of dying between age 30 and exact 70 from any of cardiovascular, cancer, diabetes, or chronic respiratory |
| x30 | Estimated antiretroviral therapy coverage among people living with HIV (%) |
| x31 | Reported number of people receiving antiretroviral therapy |
| x32 | Number of incident tuberculosis cases |
| **x33** | **Incidence of tuberculosis (per 100 000 population per year)** |
| x34 | Deaths due to tuberculosis among HIV-negative people (per 100 000 population) |
| x35 | Number of deaths due to tuberculosis, excluding HIV |
| x36 | Tuberculosis treatment coverage |
| x37 | Tuberculosis - new and relapse cases |

**Table S1** List of potential risk factors, with the 10 selected risk factors for model analysis highlighted in bold.

*Supplement S1.3: Model evaluation metric and procedure*

To address unexplained variability, random effects were incorporated into the spatiotemporal model. These random effects captured variations that could not be explained by fixed effects, including differences between countries or regions due to cultural, economic, or mental health factors that are not directly measurable. Incorporating random effects enhanced the model’s flexibility, reduced biases, and improved the reliability of the results. The spatiotemporal analysis aimed to achieve two primary objectives: identifying clusters of countries or regions with similar patterns of suicide behavior (cluster detection) and analyzing associations between these clusters and selected risk factors. The model selection process evaluated various combinations of space-time fixed and random effects using several performance metrics, including the Deviance Information Criterion (DIC), Watanabe-Akaike Information Criterion (WAIC), Root Mean Squared Error (RMSE), Conditional Predictive Ordinate (CPO), and Spearman’s correlation coefficient. Best-fitting models were identified separately for males, females, and combined sexes.

In model selection, the primary objective is to identify the simplest model that optimally fits the observed data. In hierarchical modeling, the choice of fixed and random effects, as well as space-time random effects, significantly impacts the estimated mean incidence and associations with risk factors. This process requires careful selection of the mean structure and random effect components to balance statistical rigor and epidemiological relevance. Spatiotemporal hierarchical modeling includes both fixed effects (explanatory variables) and random effects, necessitating thoughtful decisions for selecting optimal explanatory variables and a suitable random-effects structure. Poorly chosen random effects can influence fixed-effect estimates and reduce overall model quality. To identify the optimal spatiotemporal mixed structure, we applied a top-down two-step procedure [7]. In step one, we used a covariate model to determine the best random-effects structure. Once the optimal random-effects structure was identified, we refined the model by testing different sets of explanatory variables in step two. The best covariates were selected based on multiple evaluation criteria derived in the first step.

To assess the relationship between suicide incidence and national-level risk factors, we examined coefficient estimates, which represent the strength of associations, and uncertainty quantification using exceedance probability. This Bayesian equivalent of the frequentist p-value was used to evaluate the significance of associations. A coefficient was considered “significant” when its exceedance probability exceeded 0.95, corresponding to a 0.05 significance threshold. The goodness-of-fit (GOF) of the models was assessed to determine how well the data aligned with the model’s spatiotemporal structure. We applied the Deviance Information Criterion (DIC) [8], a widely used Bayesian GOF measure generalized from the Akaike Information Criterion (AIC) in the frequentist framework. Additionally, we used the Watanabe-Akaike Information Criterion (WAIC) [9], an improvement over DIC. WAIC is a fully Bayesian measure that utilizes the entire posterior distribution and is robust to different parameterizations, making it valid for singular models [10].

Other metrics for model evaluation included bias, computed as the average difference between observed incidences and model estimates across locations and time periods, with values close to zero being preferred. We also calculated the root mean squared error (RMSE), which measures the squared root of the average squared deviation between observed and estimated incidences across spatial units and study periods. Additionally, correlation was used to assess the strength of association between observed and estimated suicide rates.

The best-fitting models were identified as follows: for combined sexes and females, the optimal model included a convolution model for spatial components, random walk order 2 for temporality, and space-time interaction type 1. For males, the best model included the Besag model for spatial components, random walk order 2 for temporality, and space-time interaction type 1. These final models facilitated the detection of high-risk clusters and the identification of key risk factors influencing the spatial distribution of suicide behaviors. The modeling process emphasized data completeness, reduction of redundancy, and interpretability. The modeling steps and processes are summarized in Figure S4, which provides a visual overview of the approach.

**
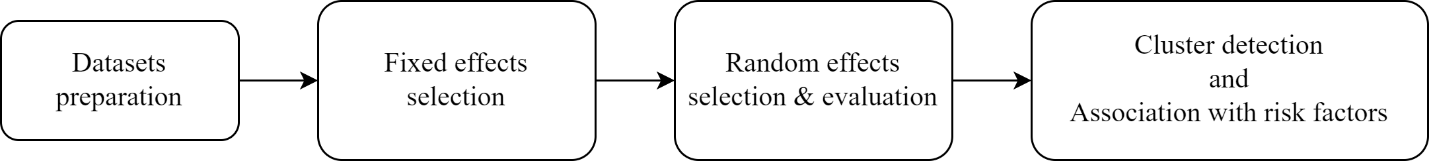
**

**Figure S4** Spatiotemporal model selection and analysis process.

**Supplementary document S2:** Global maps of country-level age-standardized and age-specific suicide rates per 100,000 population.


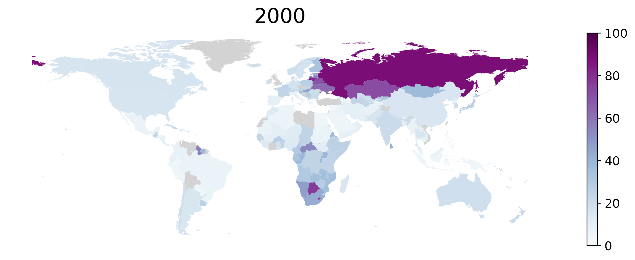

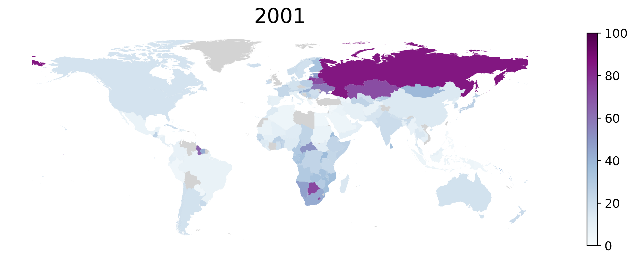

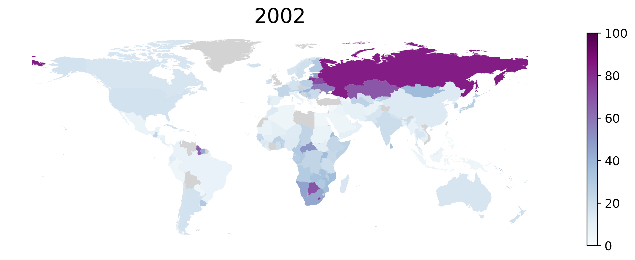

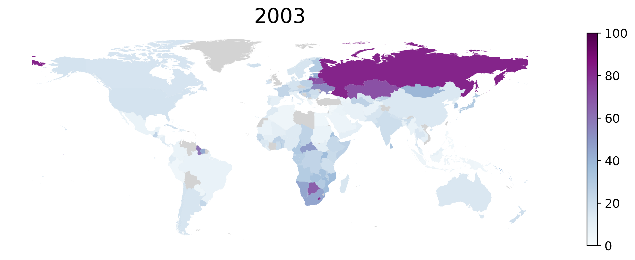

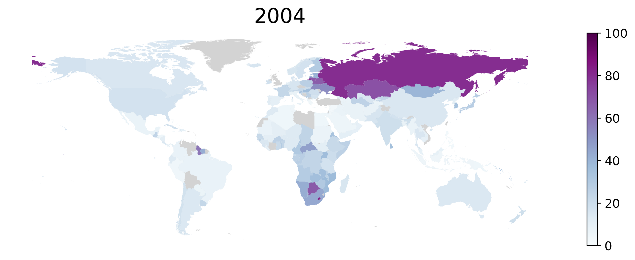

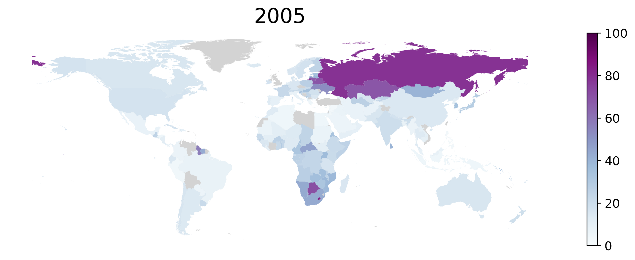

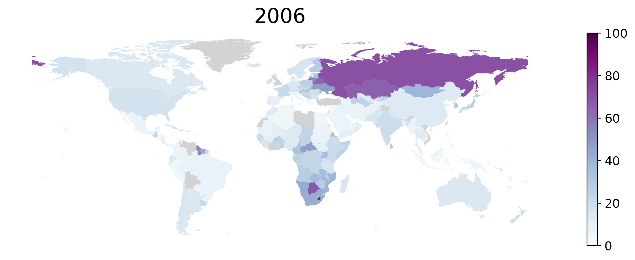

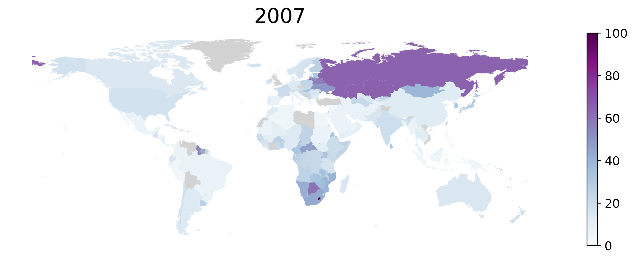

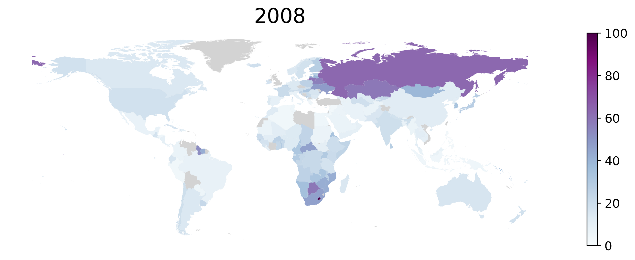

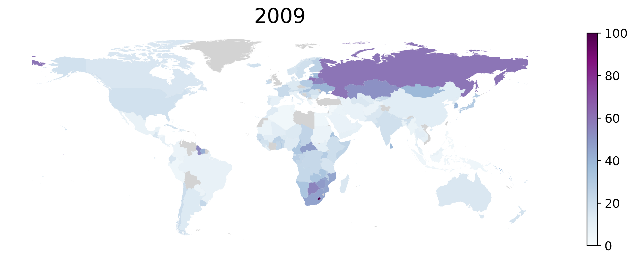

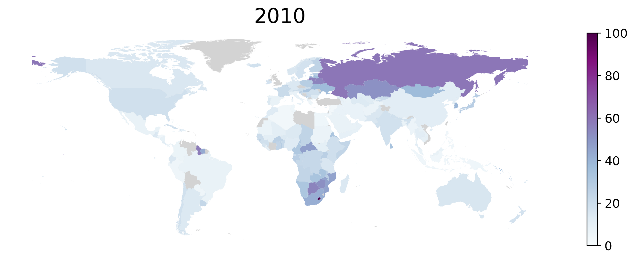

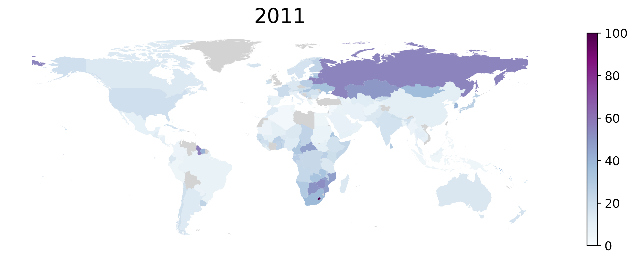

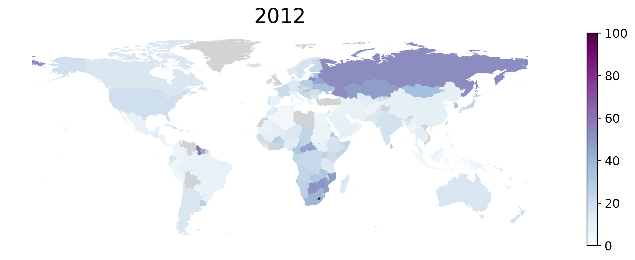

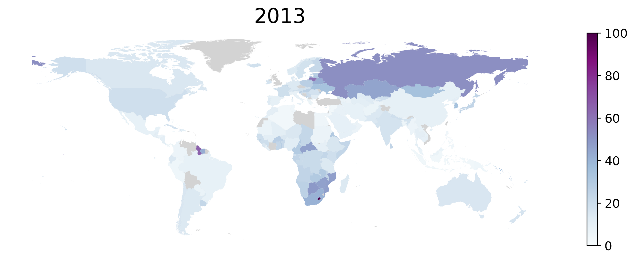

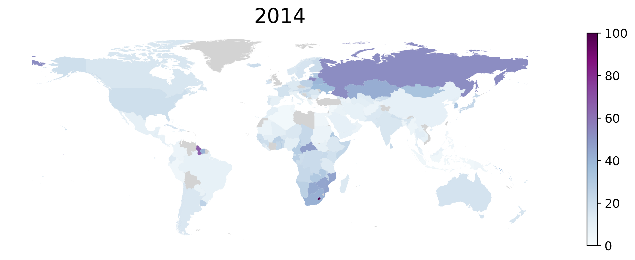

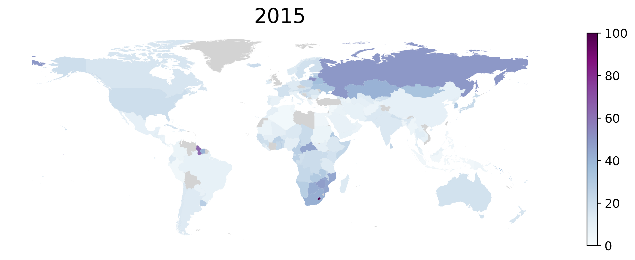


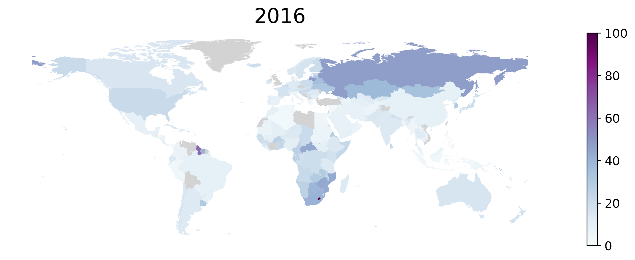

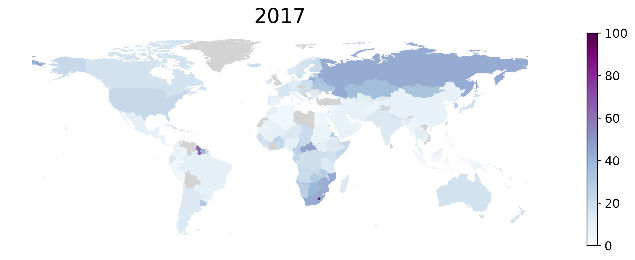

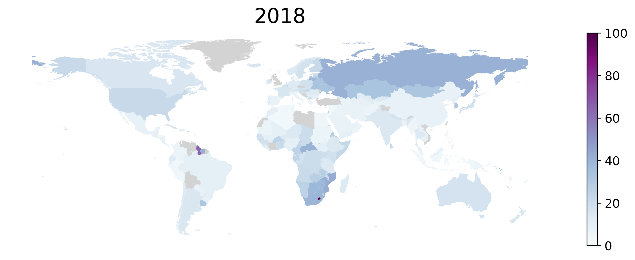

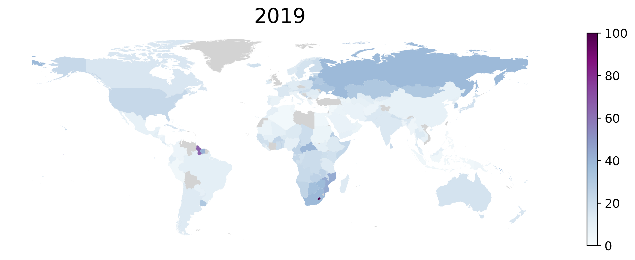


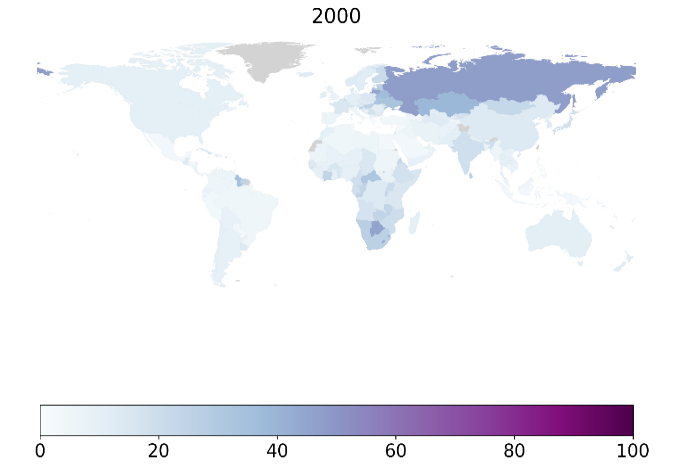


**Figure S5** Global maps of country-level age-standardized suicide rates among males (2000-2019) per 100,000 population, generated using RStudio version 2022.07.0+548 (available at https://posit.co/products/open-source/rstudio/).

.


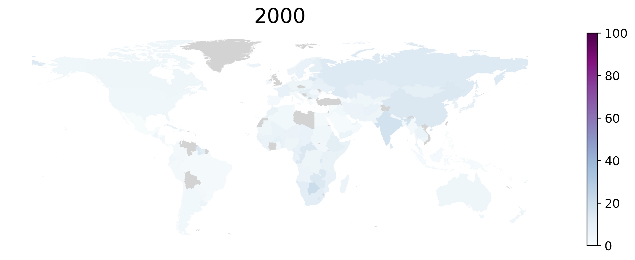

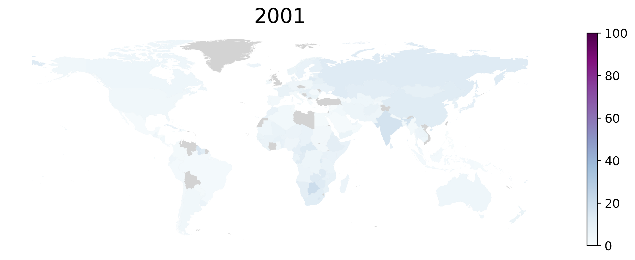

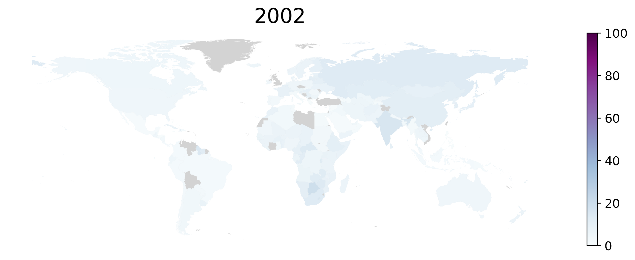

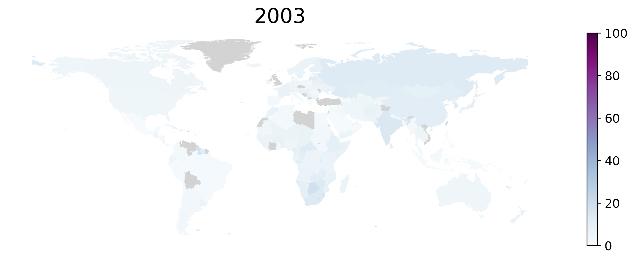

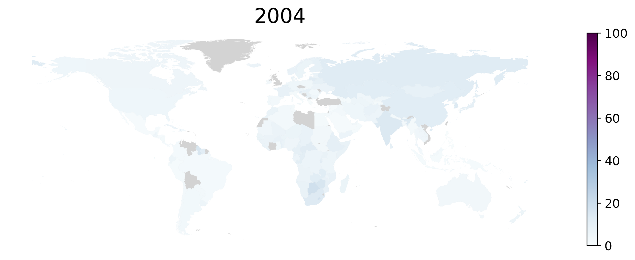

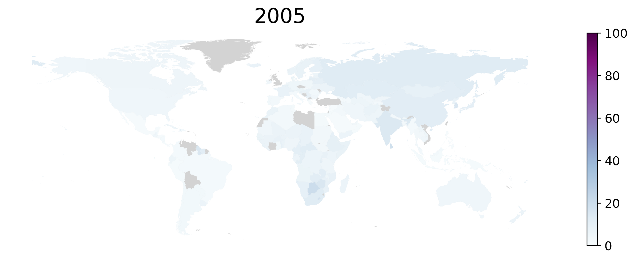

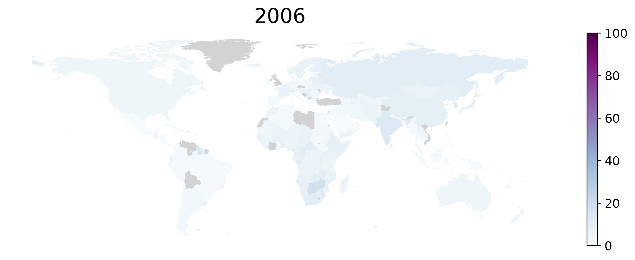

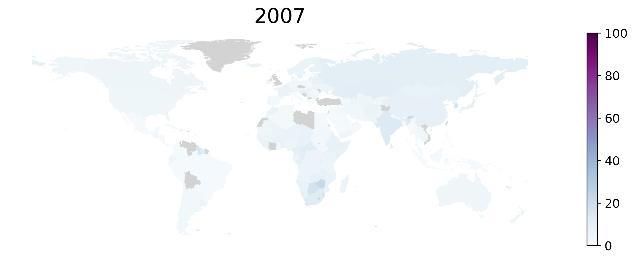


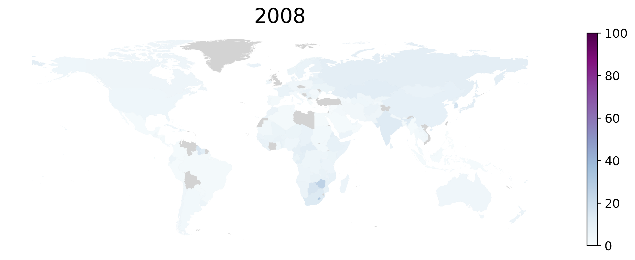

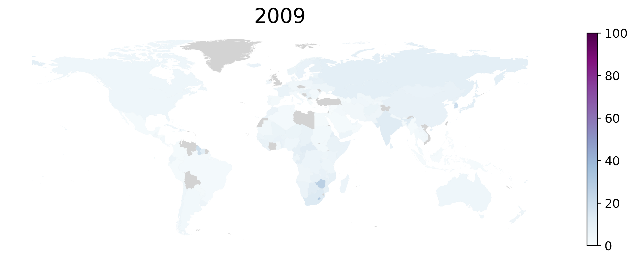

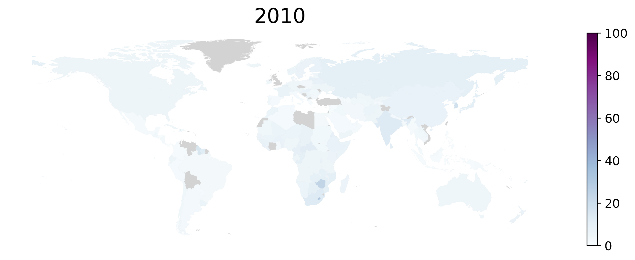

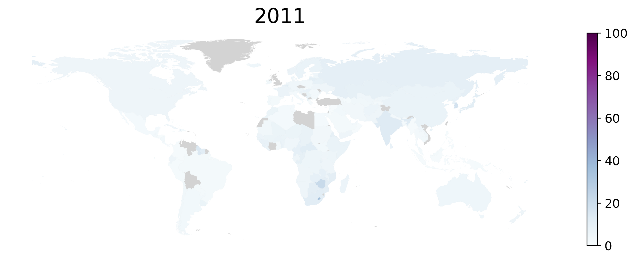

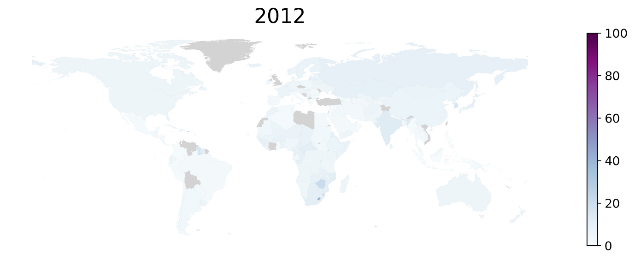

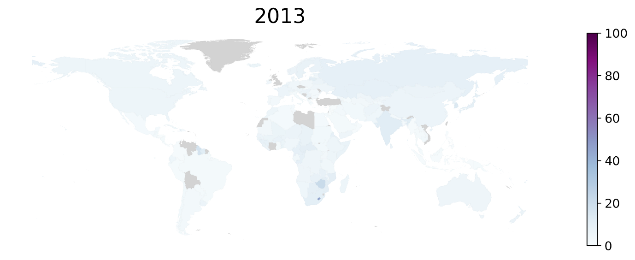

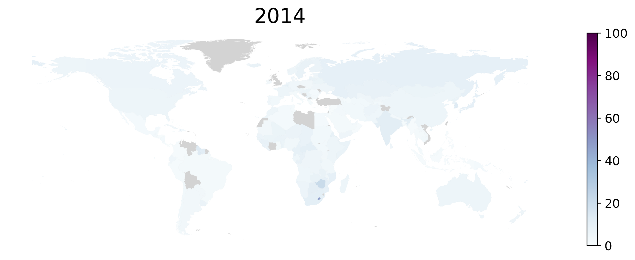

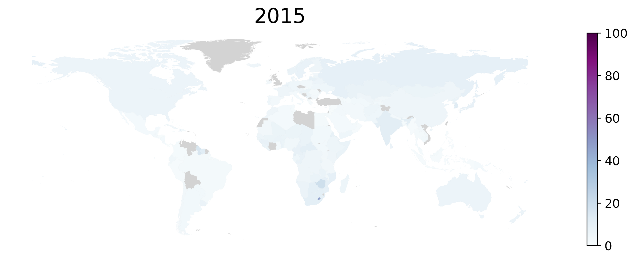

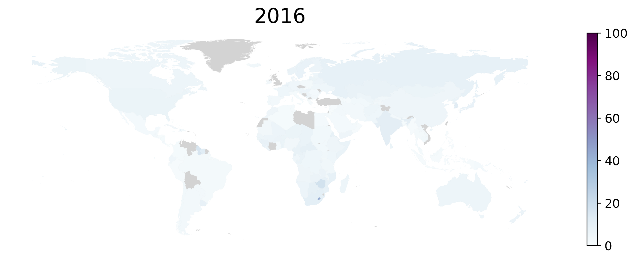

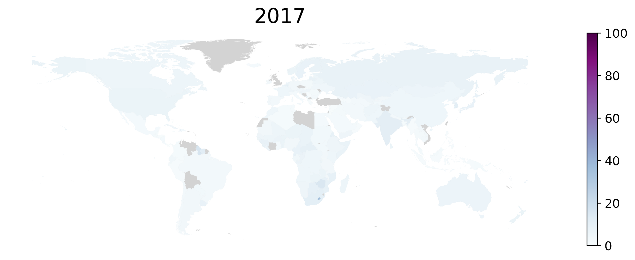

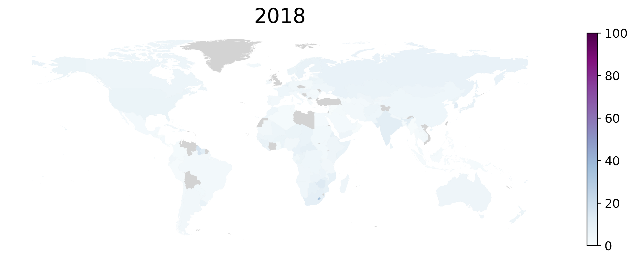

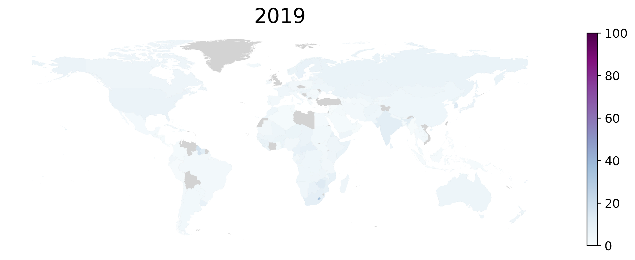


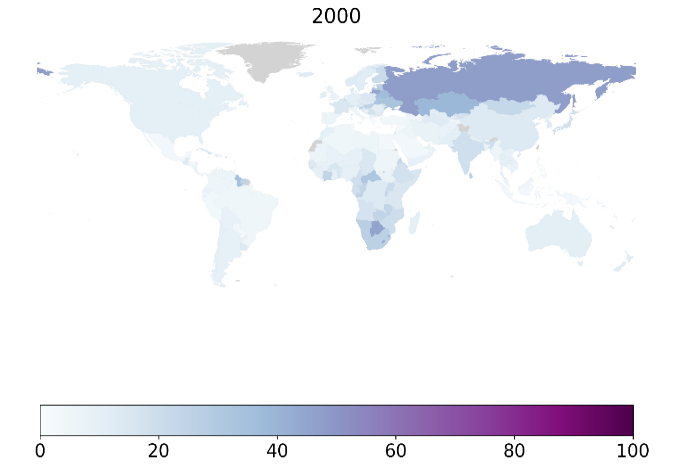


**Figure S6** Global maps of country-level age-standardized suicide rates among females (2000-2019) per 100,000 population, generated using RStudio version 2022.07.0+548 (available at https://posit.co/products/open-source/rstudio/).

Male Female


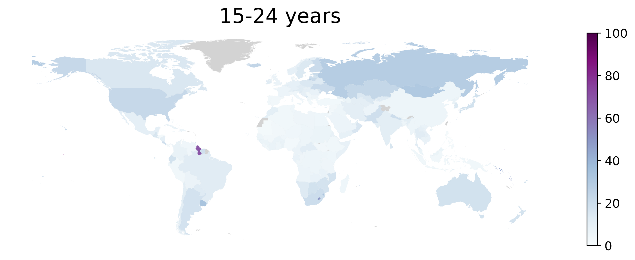

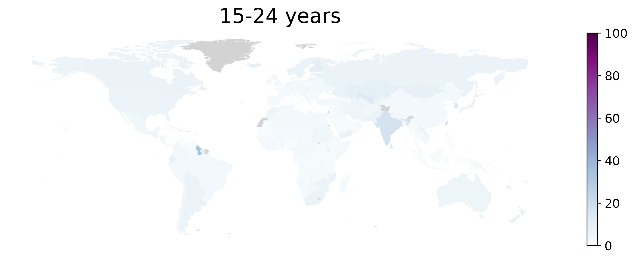


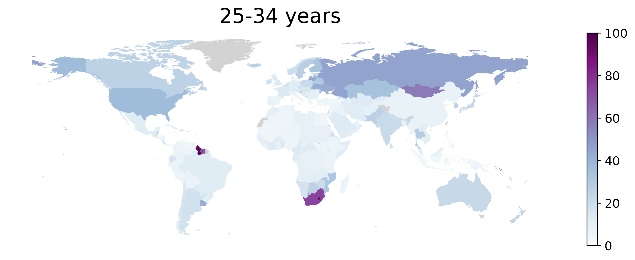

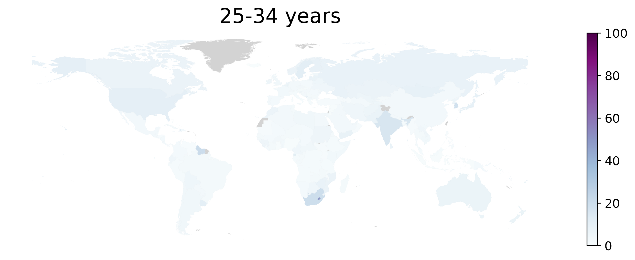


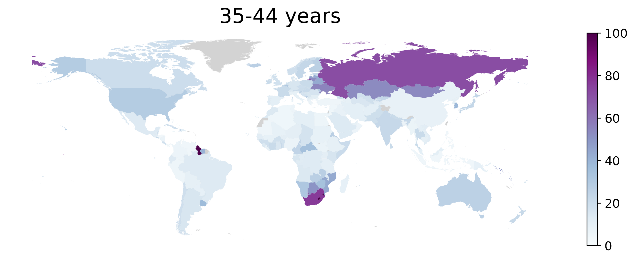

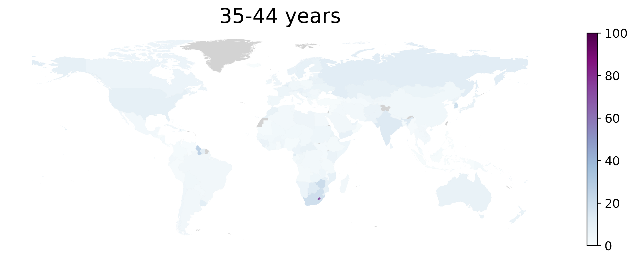


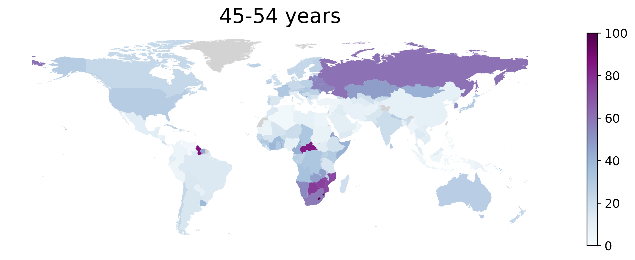

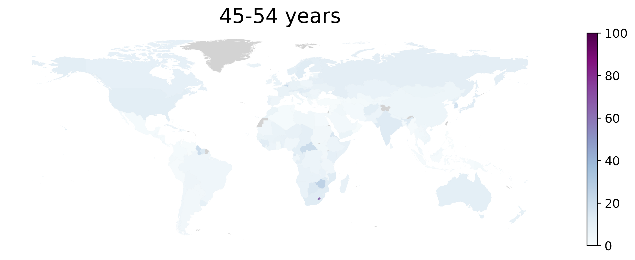


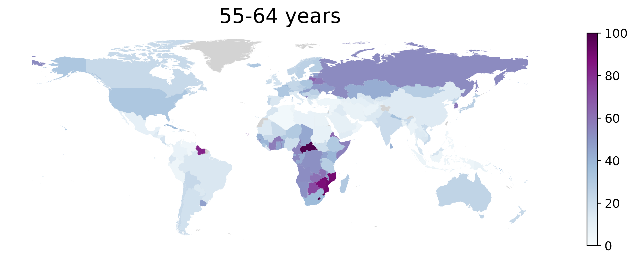

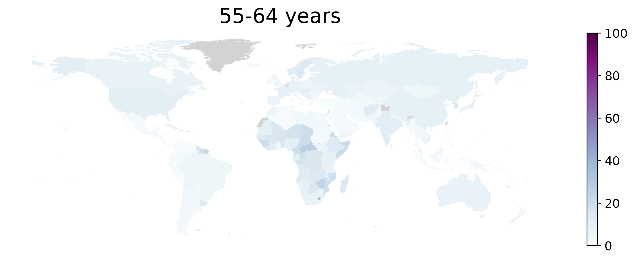


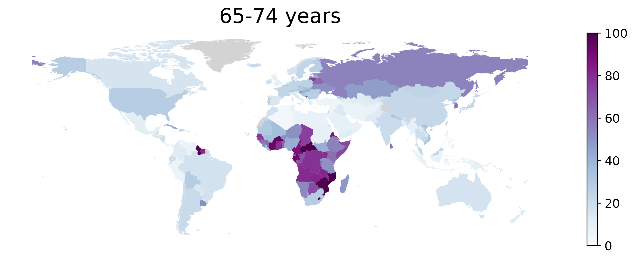

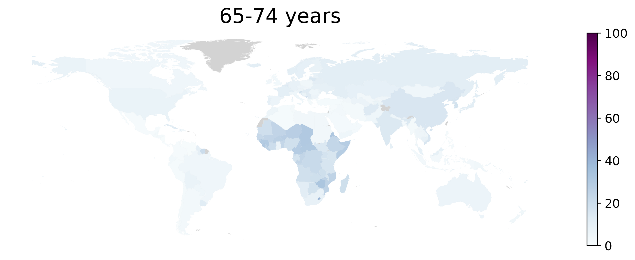


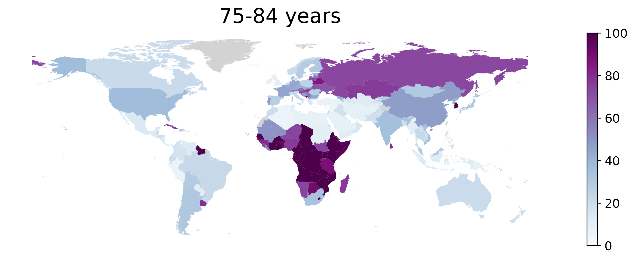

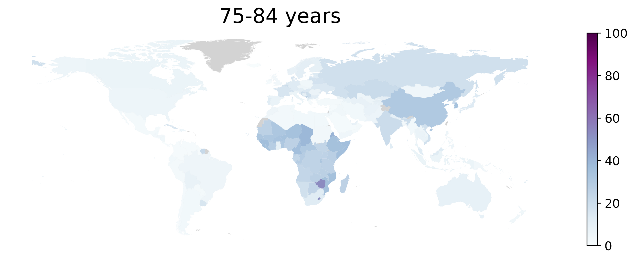


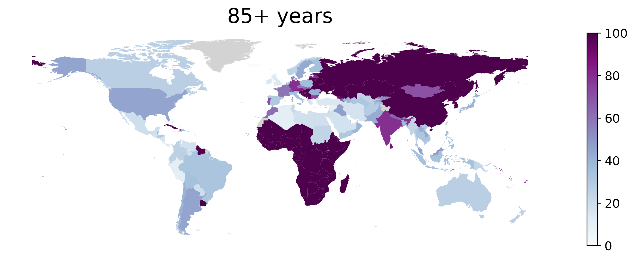

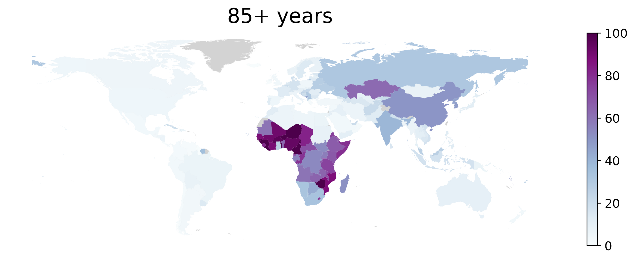


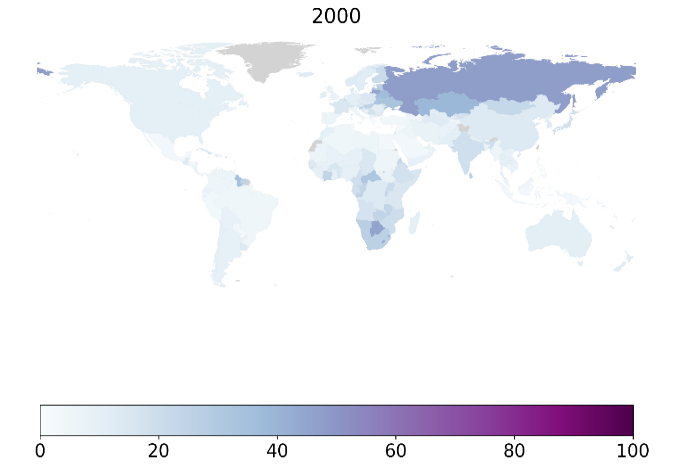


**Figure S7** Global maps of country-level age-specific suicide rates in 2019 per 100,000 population for each sex, generated using RStudio version 2022.07.0+548 (available at https://posit.co/products/open-source/rstudio/).


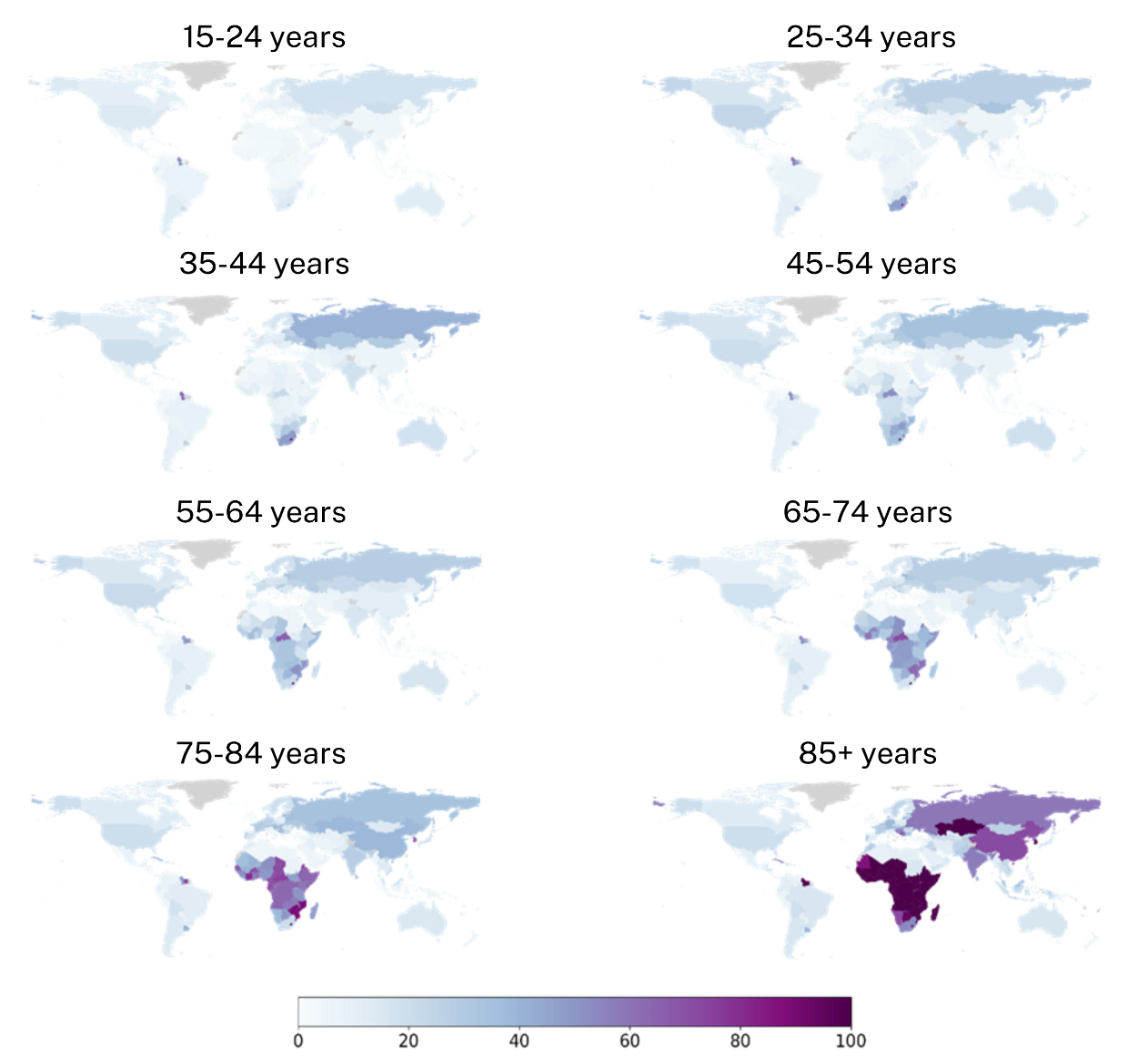


**Figure** **S8** Global maps of country-level age-specific suicide rates in 2019 per 100,000 population divided into age groups for both sexes combined, generated using RStudio version 2022.07.0+548 (available at https://posit.co/products/open-source/rstudio/).

**Supplementary document S3:** Plots of global age-standardized suicide rates by region and top five countries with highest age-standardized suicide rates in each region (2000-2019) per 100,000 population.


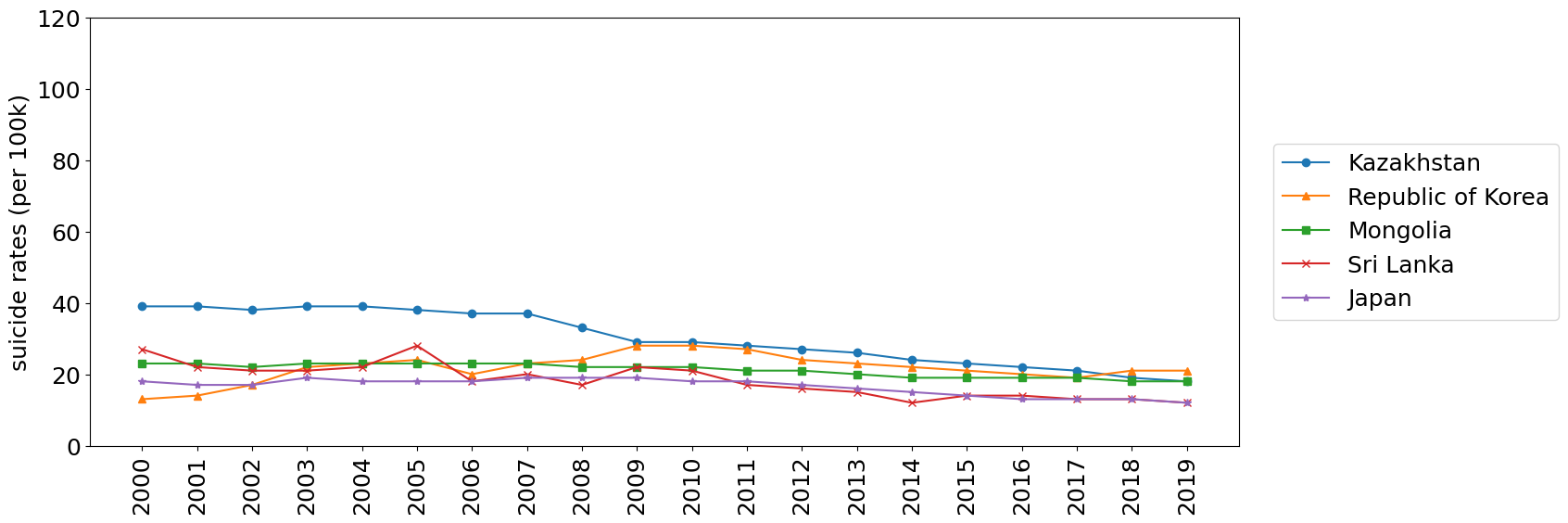


**Figure S9** Plots of top five countries with highest age-standardized suicide rates in Asia (2000-2019) per 100,000 population.


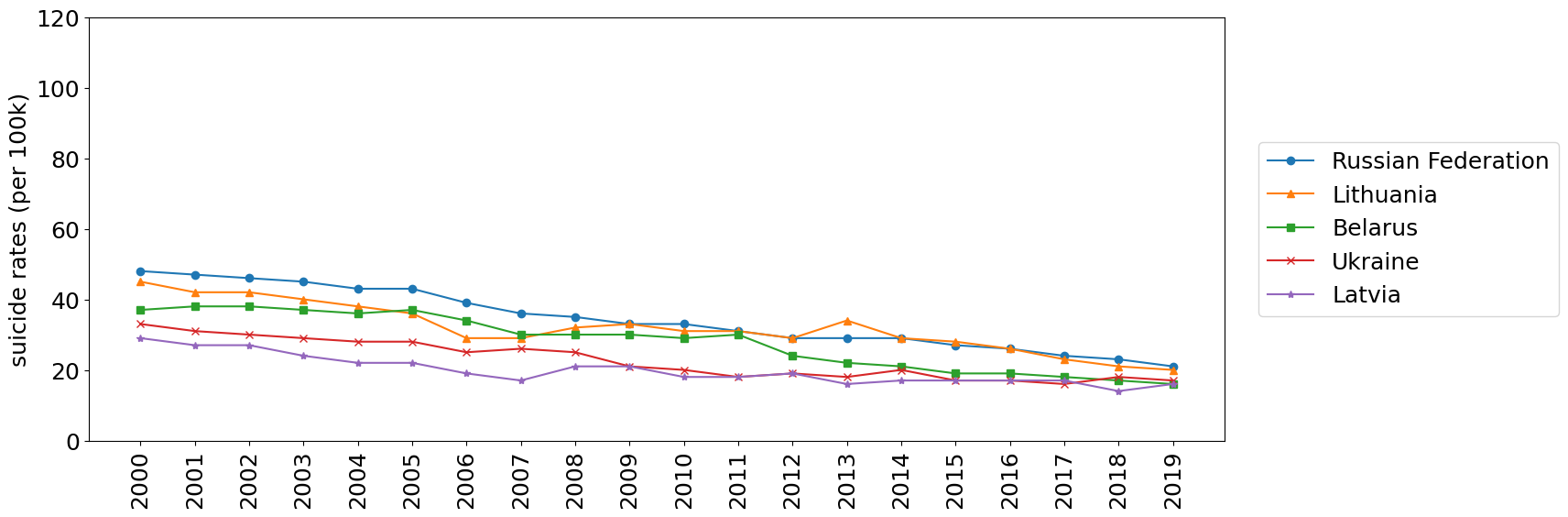


**Figure S10** Plots of top five countries with highest age-standardized suicide rates in Europe (2000-2019) per 100,000 population.


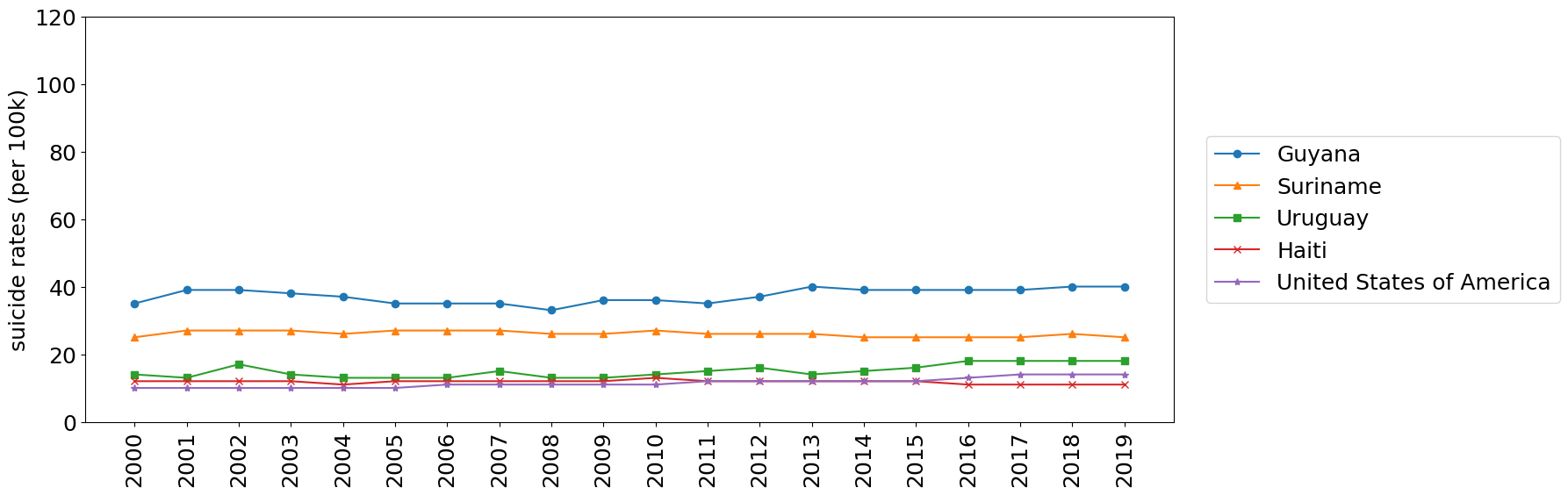


**Figure S11** Plots of top five countries with highest age-standardized suicide rates in America (2000-2019) per 100,000 population.


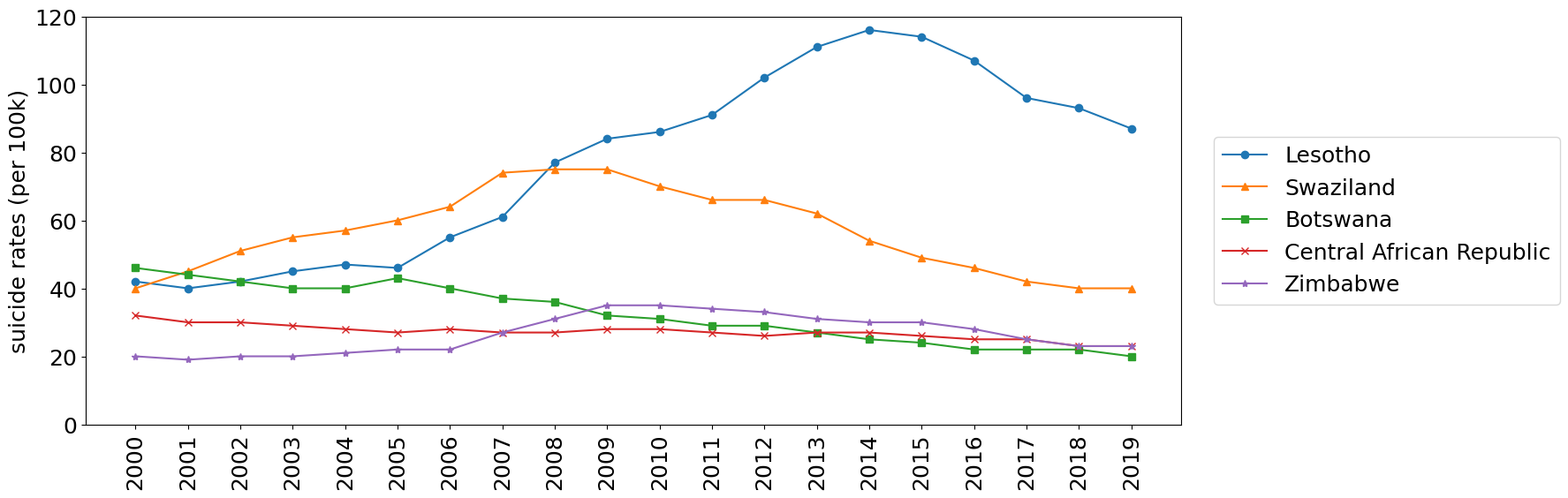


**Figure S12** Plots of top five countries with highest age-standardized suicide rates in Africa (2000-2019) per 100,000 population.


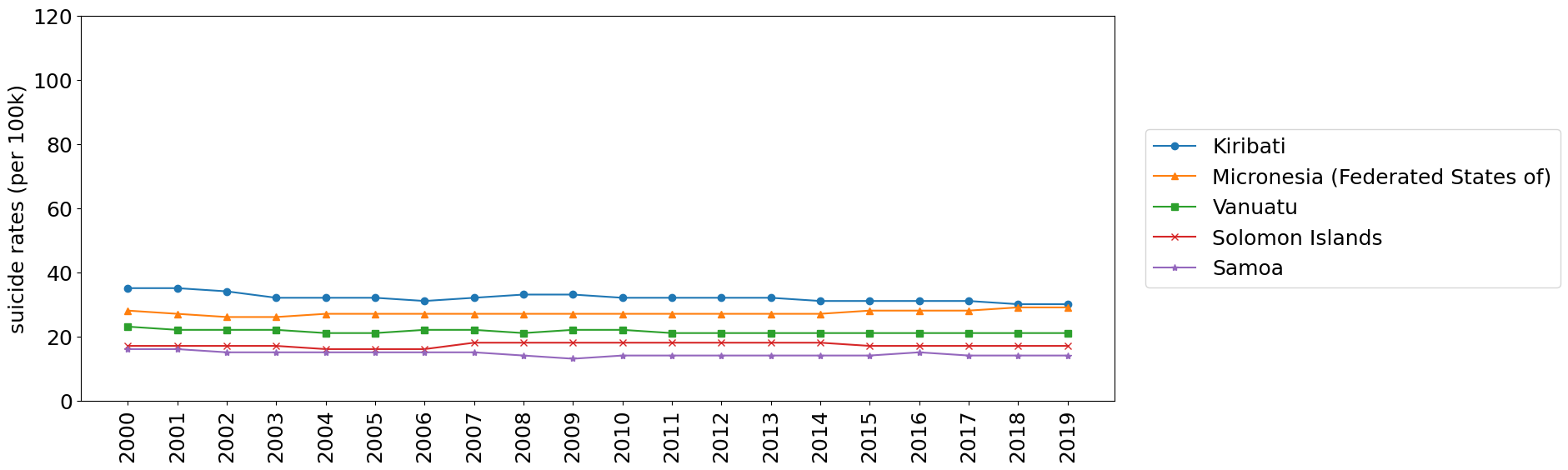


**Figure S13** Plots of top five countries with highest age-standardized suicide rates in Oceania

(2000-2019) per 100,000 population.

**Supplementary document S4:** Global maps of country-level age-standardized suicide hotspots (2000 – 2019).


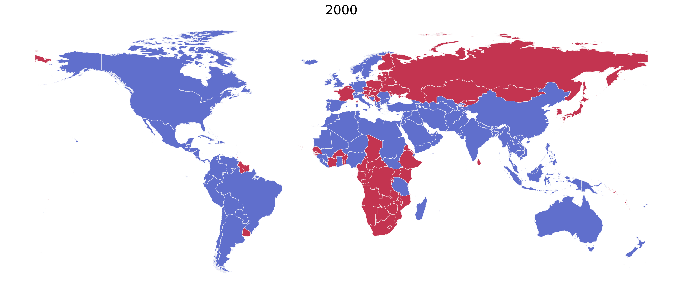
**
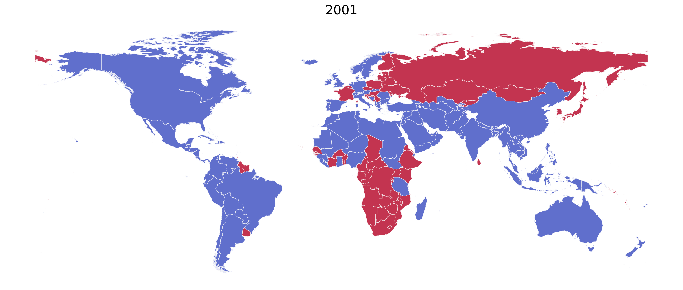

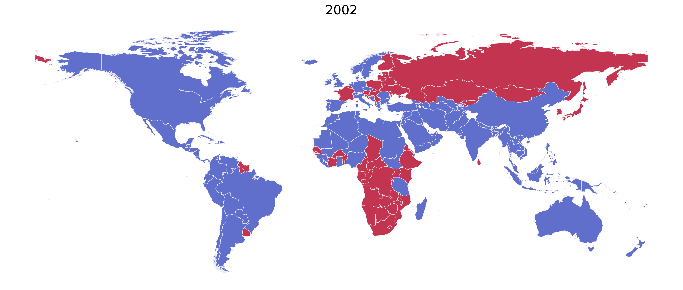

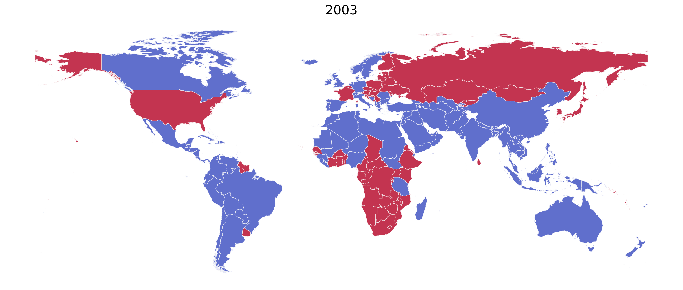

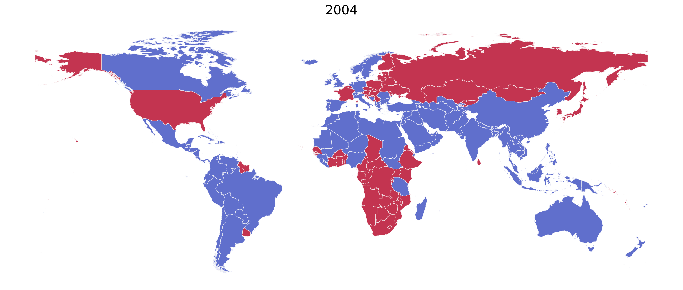

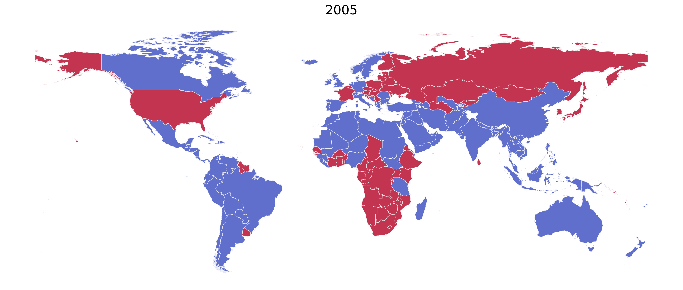

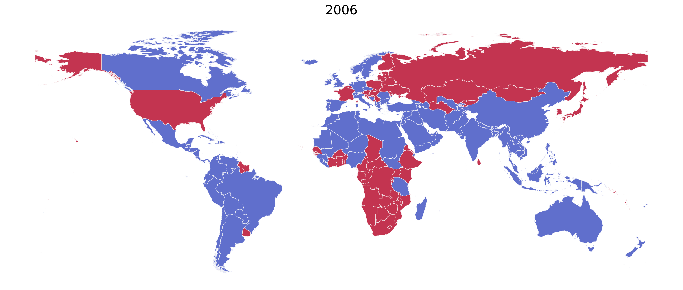

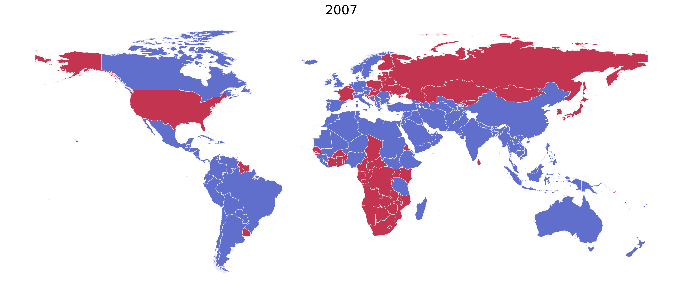

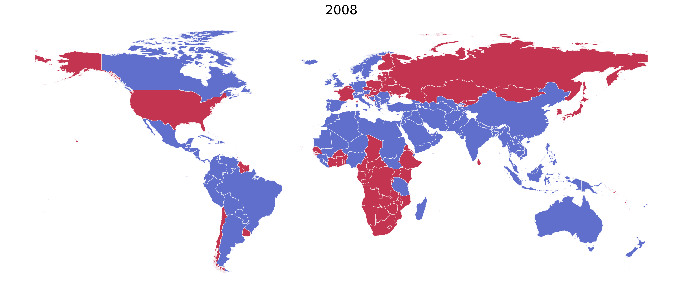

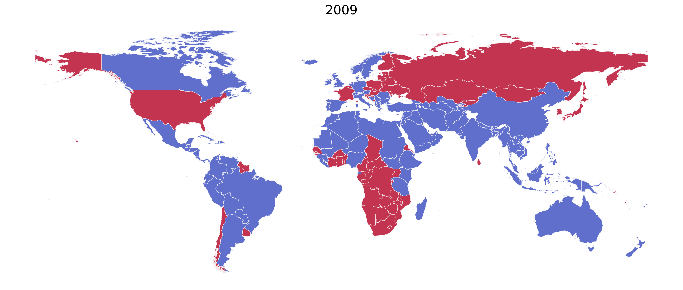

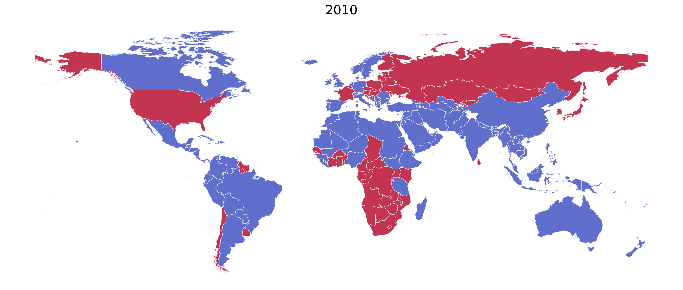

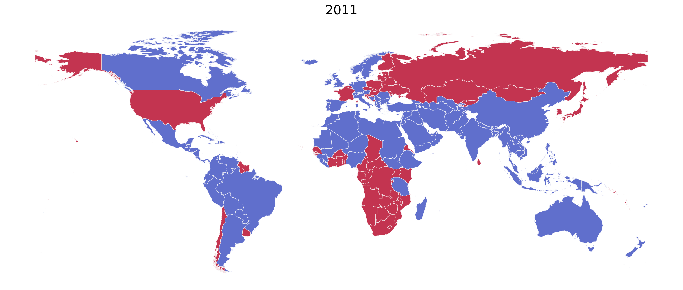

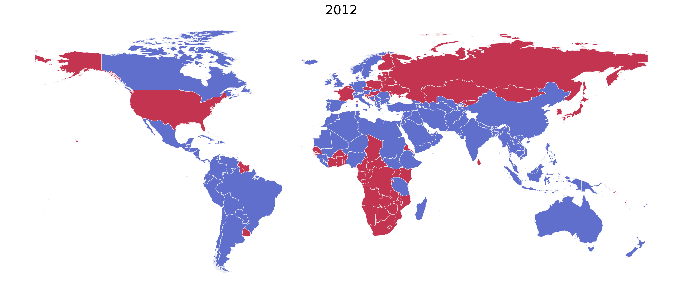

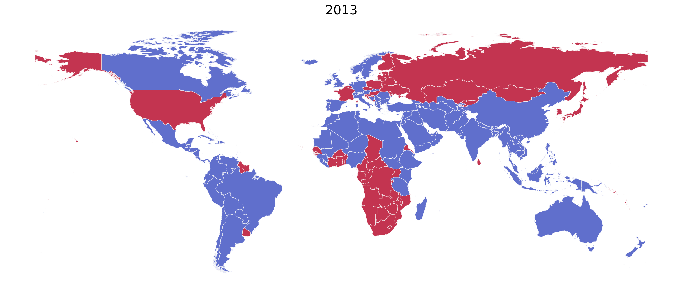

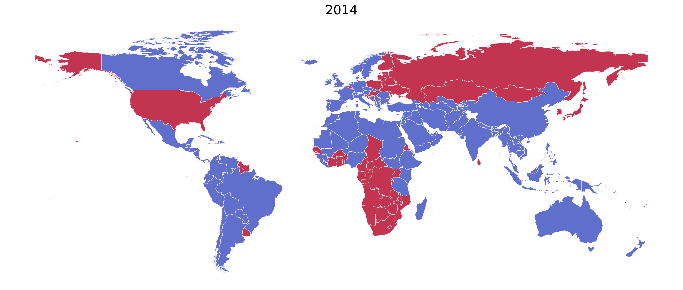

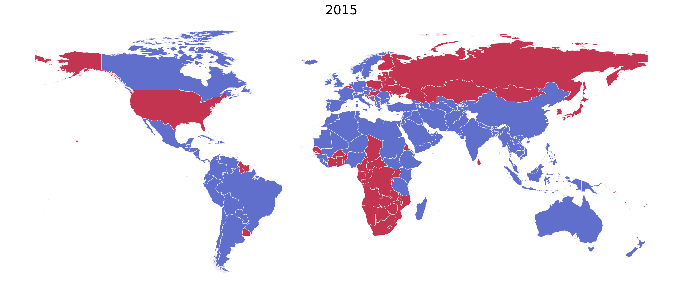

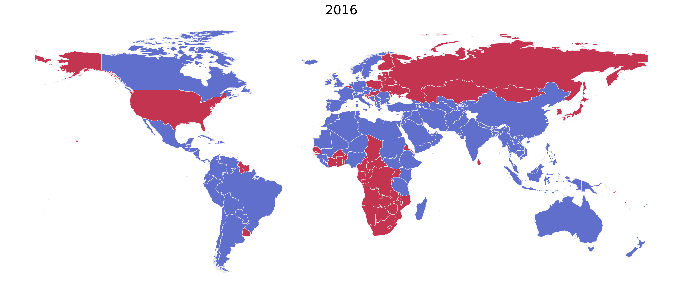

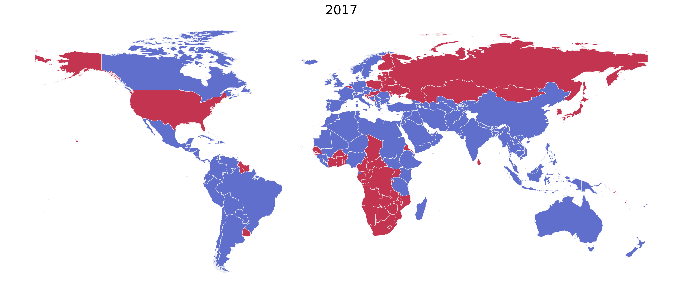

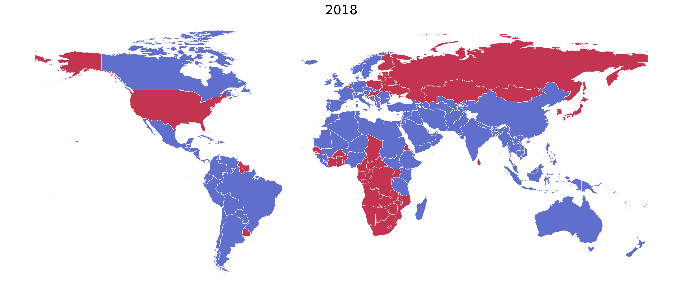

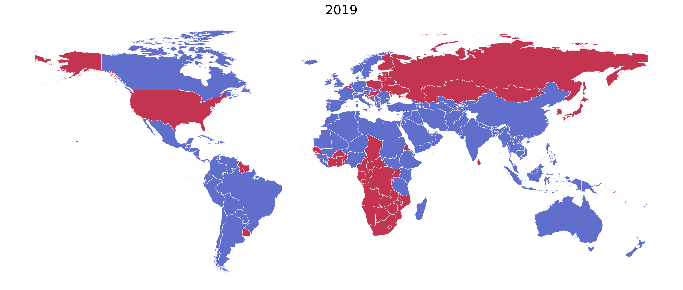
**

**
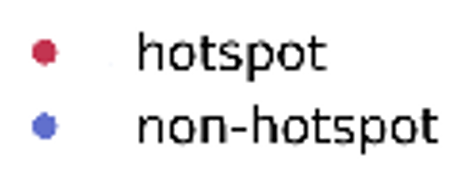
**

**Figure S14** Global maps of country-level age-standardized suicide hotspots

among males between 2000 and 2019, generated using RStudio version 2022.07.0+548 (available at https://posit.co/products/open-source/rstudio/).

**
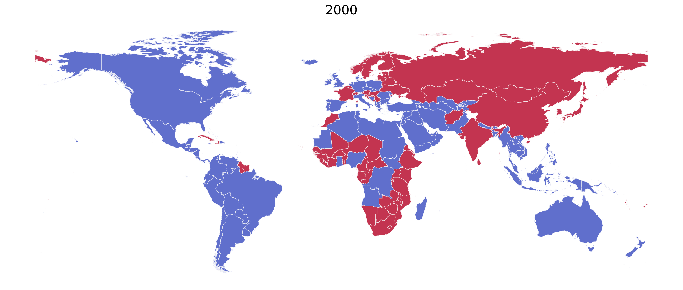

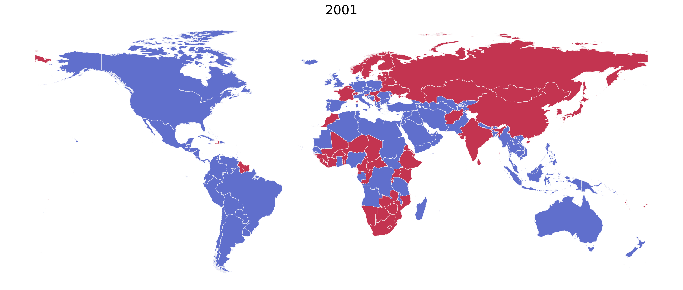

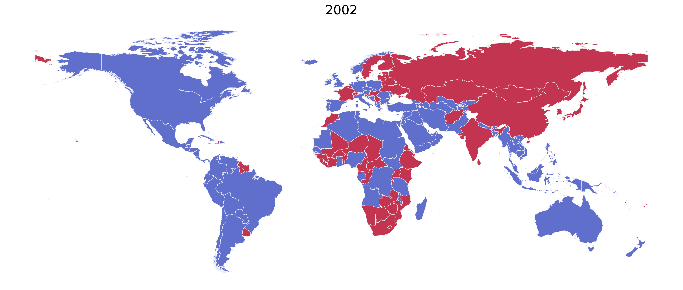

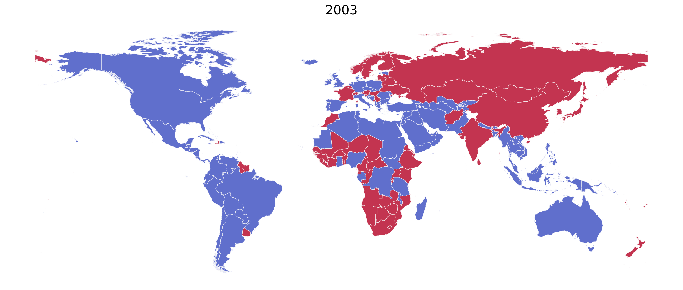

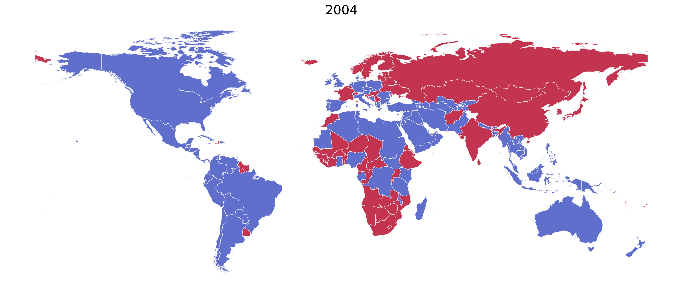

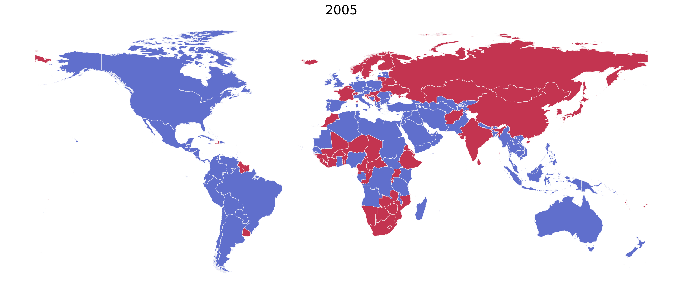

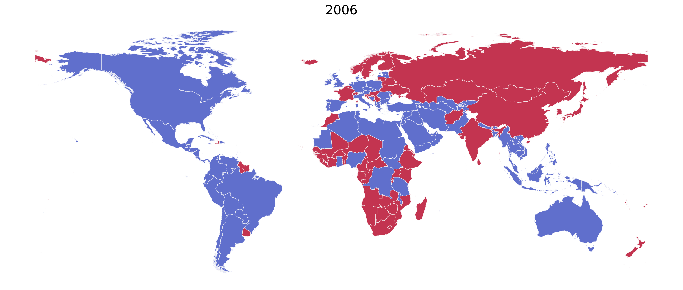

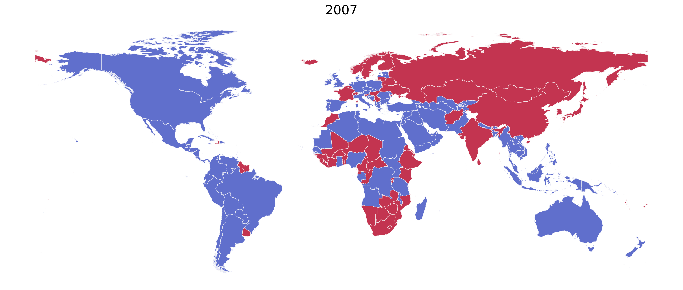

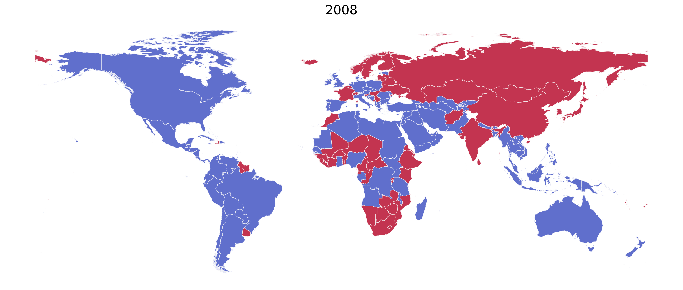

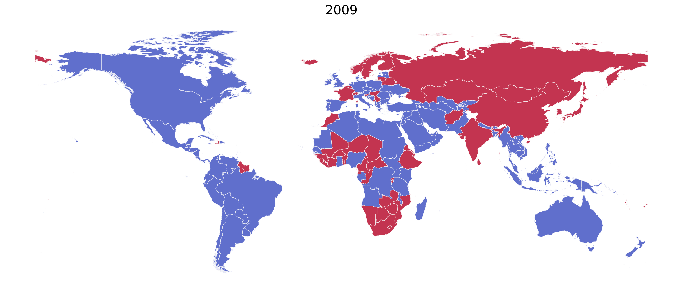
**

**Figure S15** Global maps of country-level age-standardized suicide hotspots

among females between 2000 and 2019, generated using RStudio version 2022.07.0+548 (available at https://posit.co/products/open-source/rstudio/).

**Figure S16** Global maps of country-level age-standardized suicide hotspots both sexes combined (2000 – 2019), generated using RStudio version 2022.07.0+548 (available at https://posit.co/products/open-source/rstudio/).

**Supplementary document S5:** Model evaluation for random-effect specification for suicide data and risk factors, the column header abbreviations and definition list, and the included risk factors list.

This document contains the model evaluation for random-effect specifications concerning suicide data and associated risk factors, along with a list of column header abbreviations and their definitions, as well as the included risk factors for both sexes and separately for each sex. Various combinations of space-time fixed and random effects were assessed by comparing their fit metrics. The primary criterion for model selection was based on the widely applicable information criterion to identify the optimal model configurations. For the combined-sex data and for females, the best random effects structure included the convolution model for the spatial component, a second-order random walk for the temporal component, and space-time interaction type one. For males, the optimal random effects specification was the Besag model, with a second-order random walk for temporality and space-time interaction type one.

**Table S17** Model evaluation model of suicide data for both sexes combined.

**Table S18** Model evaluation model of suicide data for males.

**Table S19** Model evaluation model of suicide data for females.

**Table S20** The column header abbreviations and definition list.

**Table S21** Included potential risk factors.

**Supplementary document S6:** Global space-time suicide association with risk factors.

When considering male suicide risk, current health expenditure (CHE) as a percentage of GDP did not show a significant association with suicide risk. On the other hand, for female suicide risk, current health expenditure (CHE) as a percentage of GDP, domestic general government health expenditure (GGHE-D) as a percentage of general government expenditure (GGE), and external health expenditure (EXT) per capita were not found to be significantly associated with suicide risk. The analysis indicated a significant association between the age-standardized non-communicable disease (NCD) mortality rate (per 100,000 population) and suicide risk in a total of 7 out of 181 countries. These associations were predominantly observed in countries located in the Americas region. In most of the countries where an association was observed, an increase in the NCD mortality rate was found to be linked to an increased risk of suicide, as indicated by the color red. These results were consistent for both male and female populations.

The domestic general government health expenditure (GGHE-D) as a percentage of CHE was found to be significantly associated with suicide risk in 4 out of 181 countries, mostly located in the Americas and Africa. In the case of males, an increase in the GGHE-D as a percentage of CHE was linked to a decrease in suicide risk in most of the associated countries. In contrast, for females, all associated countries showed an increase in suicide risk with an increase in the current health expenditure (CHE) as a percentage of GDP. The domestic general government health expenditure (GGHE-D) as a percentage of general government expenditure (GGE) was found to have a significant association with suicide risk in 4 out of 181 countries, primarily from the Americas. Among males, in the countries where an association was observed, an increase in the GGHE-D as a percentage of GGE was linked to both a decrease and an increase in suicide risk.

A significant association was found between external health expenditure (EXT) per capita in US $ and suicide risk, mainly in Africa. In the countries where this association was observed, an increase in the EXT per capita in US $ was associated with a decrease in suicide risk, as indicated by green. Among males, in the country where this association was found, an increase in the EXT per capita in US $ was also linked to a decrease in suicide risk. A significant association was found between the estimated number of road traffic deaths and suicide risk in 7 out of 181 countries, mostly in the Americas. This association was quite similar to the association between the NCD mortality rate and suicide risk. In some countries, an increase in the number of road traffic deaths was correlated with an increase in suicide risk, whereas in others, it corresponded to a decrease in suicide risk, indicated by the colour green. These results were consistent for both male and female populations.

The population using at least basic drinking-water services was found to be significantly associated with suicide risk, mostly in the Americas. Among females, in the majority of countries where this association was observed, an increase in the population using at least basic drinking-water services was associated with a decrease in suicide risk. Similarly, among males, in all countries where an association was observed, an increase in the population using at least basic drinking-water services was associated with a decrease in suicide risk. A significant association was observed in 8 out of 181 countries, mainly in the Americas, between the incidence of tuberculosis (per 100,000 population per year) and suicide risk. In some countries, an increase in the incidence of tuberculosis was associated with an increase in suicide risk, while in others, it was associated with a decrease in suicide risk, indicated by the colour green. These results were consistent for both male and female populations.

**Figure S22** Global maps of the association between risk factors and age-standardized suicide rates among males, generated using RStudio version 2022.07.0+548 (available at https://posit.co/products/open-source/rstudio/).

**Figure S23** Global maps of the association between risk factors and age-standardized suicide rates among females, generated using RStudio version 2022.07.0+548 (available at https://posit.co/products/open-source/rstudio/).

**References**

1. Besag, J., J. York, and A. Mollié, *Bayesian image restoration, with two applications in spatial statistics.* Annals of the institute of statistical mathematics, 1991. **43**(1): p. 1-20.

2. Knorr‐Held, L., *Bayesian modelling of inseparable space‐time variation in disease risk.* Statistics in medicine, 2000. **19**(17‐18): p. 2555-2567.

3. Blangiardo, M. and M. Cameletti, *Spatial and spatio-temporal Bayesian models with R-INLA*. 2015: John Wiley & Sons.

4. Cairns, J.-M., E. Graham, and C. Bambra, *Area-level socioeconomic disadvantage and suicidal behaviour in Europe: A systematic review.* Soc. Sci. Med., 2017. **192**: p. 102-111.

5. Zhang, H., et al., *Spatial analysis and risk factors of suicide among people living with HIV/AIDS who committed suicide.* Int J STD AIDS, 2021. **32**(6): p. 490-500.

6. Ratner, B., *The correlation coefficient: Its values range between+ 1/− 1, or do they?* Journal of targeting, measurement and analysis for marketing, 2009. **17**(2): p. 139-142.

7. West, B.T., K.B. Welch, and A.T. Galecki, *Linear mixed models: a practical guide using statistical software*. 2006: Chapman and Hall/CRC.

8. Spiegelhalter, D.J., et al., *Bayesian measures of model complexity and fit.* J. R. Stat. Soc. Series B Stat. Methodol., 2002. **64**(4): p. 583-639.

9. Watanabe and Opper, *Asymptotic equivalence of Bayes cross validation and widely applicable information criterion in singular learning theory.* J. Mach. Learn. Res., 2010.

10. Vehtari, A., A. Gelman, and J. Gabry, *Practical Bayesian model evaluation using leave-one-out cross-validation and WAIC.* Statistics and Computing, 2017. **27**(5): p. 1413-1432.
